# mTORC1-c-Myc pathway rewires methionine metabolism for HCC progression through suppressing SIRT4 mediated ADP ribosylation of MAT2A

**DOI:** 10.1186/s13578-022-00919-y

**Published:** 2022-11-12

**Authors:** Liang Zhao, Huizhao Su, Xiaomeng Liu, Hongquan Wang, Yukuan Feng, Yan Wang, Haiqiang Chen, Luo Dai, Shihui Lai, Siqi Xu, Chong Li, Jihui Hao, Bo Tang

**Affiliations:** 1 Department of Pancreatic Cancer, Tianjin Medical University Cancer Institute and Hospital, National Clinical Research Center for Cancer, Key Laboratory of Cancer Prevention and Therapy, Tianjin’s Clinical Research Center for Cancer, Tianjin, 300060 China; 2 Department of Hepatobiliary Surgery, The First Affiliated Hospital of Guangxi Medical University, Nanning, 530021 Guangxi People’s Republic of China; 3 Zhongke Jianlan Medical Research Institute, Beijing, 101400 China; 4 Institute of Biophysics, Chinese Academy of Sciences, Beijing, 100101 China

**Keywords:** Cancer, mTOR, c-Myc, SIRT4, MAT2A, TRIM32, ADP ribosylation, Methionine metabolism

## Abstract

**Background:**

Exploiting cancer metabolism during nutrient availability holds immense potential for the clinical and therapeutic benefits of hepatocellular carcinoma (HCC) patients. Dietary methionine is a metabolic dependence of cancer development, but how the signal transduction integrates methionine status to achieve the physiological demand of cancer cells remains unknown.

**Methods:**

Low or high levels of dietary methionine was fed to mouse models with patient-derived xenograft or diethyl-nitrosamine induced liver cancer. RNA sequence and metabolomics were performed to reveal the profound effect of methionine restriction on gene expression and metabolite changes. Immunostaining, sphere formation assays, in vivo tumourigenicity, migration and self-renewal ability were conducted to demonstrate the efficacy of methionine restriction and sorafenib.

**Results:**

We discovered that mTORC1-c-Myc-SIRT4 axis was abnormally regulated in a methionine-dependent manner and affected the HCC progression. c-Myc rewires methionine metabolism through TRIM32 mediated degradation of SIRT4, which regulates MAT2A activity by ADP-ribosylation on amino acid residue glutamic acid 111. MAT2A is a key enzyme to generate S-adenosylmethionine (SAM). Loss of SIRT4 activates MAT2A, thereby increasing SAM level and dynamically regulating gene expression, which triggers the high proliferation rate of tumour cells. SIRT4 exerts its tumour suppressive function with targeted therapy (sorafenib) by affecting methionine, redox and nucleotide metabolism.

**Conclusions:**

These findings establish a novel characterization of the signaling transduction and the metabolic consequences of dietary methionine restriction in malignant liver tissue of mice. mTORC1, c-Myc, SIRT4 and ADP ribosylation site of MAT2A are promising clinical and therapeutic targets for the HCC treatment.

**Supplementary Information:**

The online version contains supplementary material available at 10.1186/s13578-022-00919-y.

## Background

Hepatocellular carcinoma (HCC) is the most common and deadly form of liver cancer worldwide. Surgical resection and liver transplantation are potential cures, but these HCC patients suffered from rapid postoperative recurrence and shortage of liver donors. Few therapeutic drugs are available as liver cancer were usually diagnosed at the later stage with the tumours resistant to conventional chemotherapy. As a result, first-line drugs show unsatisfactory efficacy, for example, sorafenib only endows three-month survival benefit over placebo [1]. Dietary restriction of essential amino acids, such as methionine, remains one of the most robust non-genetic interventions to date for cancer treatment [2–12], including hepatocellular carcinoma [5, 6, 10]. Intriguingly, tumour initiating cells are highly dependent on methionine consumption, and the tumour volume is highly corelated with intra-tumour methionine levels [3, 11]. Dietary methionine restriction has been shown to retard tumour progression and sensitize cancer chemotherapy in the mouse model [2, 5, 8, 9]. Hepatocyte nuclear factor 4α regulated hepatic sulfur amino acid metabolism and the sensitivity of liver cancer to methionine restriction [5]. However, the underlying mechanism about how the dietary methionine reprograms cellular metabolism and its subsequent consequences on cancer progression is still not clear.

The mammalian target of rapamycin complex 1 (mTORC1) is a central sensor of amino acid signaling [13, 14]. mTORC1 signaling regulates a variety of metabolic pathways through activating transcription factors, including an oncogenic transcriptional factor c-Myc [14, 15]. c-Myc contributes to 30–50% of human malignancies by regulating up to 15% of human genes. c-Myc reprograms cellular metabolism to sustain the high proliferation rate of cancer cells. However, little is known about the role of c-Myc in the modulation of methionine metabolism.

Sirtuin 4 is one of highly conserved NAD^+^-dependent protein deacylases that regulate post-translational modifications of various proteins [16, 17]. SIRT4 possesses deactylation, lipoamidase and adenosine diphosphate (ADP)/ribosyltransferase activity to regulate catabolism of multiple nutrients, including fatty oxidation, lipid and amino acids [18–25]. SIRT4 has been shown to act as an ADP-ribosyltrasferase to ADP-ribosylate glutamate dehydrogenase, which promotes the metabolism of glutamate and glutamine [18, 24]. Whether SIRT4 regulates other amino acid metabolism by modulating ADP-ribosylation of enzymes is yet not known.

Given that methionine is a crucial fuel for tumour cells, increased methionine cycle is critical for driving tumorigenesis [3]. Methionine adenosyltransferase (MAT) consists of two distinct genes, MAT1A and MAT2A. In hepatocellular carcinoma, the dominant isoform MAT1A is downregulated while the extrahepatic isoform MAT2A is highly expressed [26, 27]. MAT2A expression has been reported to be regulated by post-transcriptional RNA methylation, which induces efficient splicing and stabilizes mRNA in response to methionine restriction [28, 29]. The activity of MAT2A could be regulated at the post-translational level. Reduced lysine acetylation at MAT2A residue 81 promoted cell proliferation in HCC [26], while MAT2A sumoylation protected cancer cells against 5-fluorouracil (5-FU) induced apoptosis [30].

Here, we investigated the role of mTORC1-c-Myc-SIRT4 axis in the regulating methionine metabolism by using tumour derived cell lines, mouse models and human specimens. First, we found that c-Myc acted as downstream of mTORC1 signaling pathway in response to methionine. Next, SIRT4 was greatly reduced by c-Myc activation, which led to reduced ADP ribosylation modification in MAT2A. Moreover, activated MAT2A promoted methionine metabolism to generate SAM for the histone methylation, leading to epigenetic modulation of gene expression. Finally, we demonstrated that methionine restriction, combined with pharmacological agents, significantly repressed HCC tumour growth in the mouse models.

## Methods

### Mouse strains

SIRT4 and c-Myc KO mice were purchased from the Jackson Laboratory. According to the guidelines, all mice were raised in filter-topped cages and fed with autoclaved food and water at Guangxi Medical University (Nanning, China). Mice were randomly divided into two groups, with one group fed a standard diet (0.86% methionine, w/w) and the other fed a methionine-restricted diet (0.12% methionine, w/w). Diethyl-nitrosamine (DEN; Sigma-Aldrich) was injected intraperitoneally into 4-week-old male mice at a single dose of 100 mg/kg. Mice were fed a diet containing 0.07% phenobarbital (Pb) from 6 weeks of age until sacrifice and executed after 8 months of DEN treatment. The development of liver cancer in mice was compared, and mice liver cells were collected for in vitro experiments. All animal experiments were reviewed and approved by the Ethics Review Committee of Guangxi Medical University.

### Cell lines

Hepatocellular carcinoma cell SNU449 and HEK293 were purchased from the ATCC (American Type Culture Collection, ATCC). Primary liver cells were isolated from fresh liver tissues. All the above cell lines were cultured in Dulbecco's modified eagle medium (DMEM, Biological Industries, Cat. No. 06-1055-57-1A) supplemented with 10% fetal bovine serum (Biological Industries, Cat. No. 04-007-1A) and 1% antibiotic/antifungal solution (Biowest, Nuaille, France). Cells were cultured in an incubator at 37 °C with 5% CO_2_ and saturated humidity.

### Antibodies

SIRT4 antibody (sc-135797) and c-Myc antibody (sc-398624) were purchased from Santa Cruz Biotechnology. β-Actin (ab8227), TRIM32 (ab96612), H3K4me3 (ab8580), H3K36me3 (ab9050), H3K4me2 (ab32356), p-mTOR (ab131538), HK2 (ab227198), SHMT1 (ab186130), GLUT1 (ab150299), ODC (ab97395) and MAT2A (ab154343) were purchased from Abcam. FLAG (CST, 2368), HA (CST, 3724), H3 (CST, 4499), H3K9me2 (CST, 9753), H3K9me3 (CST, 13969), H3K27me3 (CST, 9733), p-S6K (CST, 9209) and S6K (CST, 9202) were purchased from Cell Signaling Technology. α-pan-ADP-Ribose binding reagent (MABE1016) was purchased from Merck.

### Cell transfection

c-Myc shRNA (sc-29226-SH), TRIM32 siRNA (sc-61714), TRIM32 shRNA (sc-61714-SH), SIRT4 shRNA (sc-63024-SH), MAT2A shRNA (sc-106203-SH) were purchased from Santa Cruz Biotechnology. Forty-eight hours later, the cells were harvested for further experiments. Cell transfections were performed using Lipofectamine 3000 (Invitrogen, Carlsbad, USA). The knockdown efficiency was confirmed by Real-time PCR and western blot.

The full-length human c-Myc, SIRT4, TRIM32 and MAT2A sequences were cloned into the pcDNA3.1 vector with or without a FLAG- or HA-tag sequence (Invitrogen, Carlsbad, USA). The siRNA and pcDNA3.1 were transfected into cells using Lipofectamine 3000 (Invitrogen, Carlsbad, USA). Forty-eight hours later, the cells were harvested for further experiments. The knockdown efficiency was confirmed by Real-time PCR and Western blot. The lentivirus shRNAs or negative control were delivered by lentiviral infection with lentiviruses produced by transfection of HEK293 cells. Cells infected with lentiviruses delivering scrambled shRNA were used as negative control cells. To select stably transfected cells, the cells were resuspended and cultured in the presence of puromycin (2 μg/ml) for 2 weeks. Real-time PCR and Western blot were performed to determine the level of gene expression.

MAT2A point mutation was performed using QuickMutation™Plus Site-Directed Mutagenesis Kit (Beyotime, Shanghai).

### Ubiquitination detection assay

FLAG-SIRT4 was co-transfected into cells with either HA-Ub, c-Myc/TRIM32 shRNA or c-Myc shRNA/TRIM32. Cells were lysed with lysis buffer. The samples were then incubated with specific anti-FLAG antibodies and Protein G Sepharose beads. Bound proteins were eluted by boiling samples in 1% SDS for 15 min and collected by centrifugation at 13,000 rpm for 5 min. Supernatants were subjected to a second round of immunoprecipitation, followed by SDS-PAGE and Western blotting as described above. To detect ubiquitinated proteins, western blots were incubated with anti-HA antibody.

### MAT2A activity assay

MAT2A activity were measured by using MAT2A assay kit according to the manufactural instructions. Briefly, 10 ul 5× buffer (250 mM TES buffer, 250 mM KCl, 75 mM MgCl_2_, 1.5 mM EDTA, 0.5 mg/ml BSA solution), 5ul 50 mM ATP, 1ul 1 mM ^14^C-L-methionine (Perkin Elmer, NEC363050UC, USA) and 0.2ul 1 M DTT was mixed together and diluted with deionized water. After incubation at 37 °C for 5 min, 40 ul mixture was mixed with a 10ul sample. After 20 min, the reaction solution (20 ul) in the tube was taken out and spot on 2 cm^2^ P81 ion exchange paper that was treated with glacial acetic acid. After being rinsed with running water for 30 min in a large container, the radioactivity of 14C-L-methionine was measured. One unit of MAT activity was defined as the amount of enzyme that catalyzes the formation of 1 nmol/ml SAM in 1 h.

### Metabolomic analysis by LC–MS/MS

Metabolites were extracted from samples by adding ice-cold extraction solution (methanol/acetonitrile/H_2_O: 2:2:1, v/v) and sonicated for 30 min in ice-water bath. After incubation for 10 min at − 20 °C, the samples were centrifuged at 14 000*g* for 20 min at 4 °C. The supernatant was withdrawn, dried and redissolved in a solution of methanol: acetonitrile: water (2:2:1, v/v/v) for the Liquid Chromatograph tandem Mass Spectrometry (LC–MS/MS) analysis. LC–MS/MS analyses were performed using an UHPLC system (Infinity LC, Agilent 1290) with a HILIC column coupled to Triple TOF 6600 mass spectrometer (Applied Biosystems SCIEX, Foster City, CA, USA).

The raw data were converted to the mzXML format using ProteoWizard and processed with an in-house program, which was developed using R and based on XCMS, for peak detection, extraction, alignment, and integration. Then an in-house MS2 database (BiotreeDB) was applied in metabolite annotation. Data reliability was checked using a quality control sample.

### In vivo tumor growth assay

Six-week-old male BALB/c nude mice were obtained (Shanghai Slac Laboratory Animal Co. Ltd., China) and bred under specific pathogen-free conditions. Treat cancer cells or control cells were subcutaneously injected into the flank regions of the mice (5 mice/group). Over a period of 6 weeks, tumour formation in the mice was observed by measuring the tumour volume. Then, the tumours were excised and weighed. All animal experiments were reviewed and approved by the Ethics Review Committee of Medical University.

SNU449, or primary liver stable cell lines with SIRT4 knockdown, MAT2A knockdown, MAT2A(E111A) mutant, c-Myc knockdown were prepared. In all, 2 × 10^6^ cells in PBS were subcutaneously injected into the flank regions of the mice (6 mice/group). The tumour volume was measured weekly over a period of 3–5 weeks, and calculated using the formula V = 0.5 × W^2^ × L (V, volume; L, Length; W, Width). Finally, the mice were sacrificed and the tumours were excised and weighed.

For PDX models of HCC cancer, HCC tumours were resected, washed and minced, and then passaged through JAX NOD. CB17-PrkdcSCID-J mice 2–5 times. For the dietary studies, HCC PDX tumours cut into 3–4 mm pieces and subcutaneously transplanted into the flanks of the animals 4 h after surgical removal. Mice were subjected to control methionine-restricted diet, either two weeks before the tumour injection or from when the tumour was palpable until the end point. Tumour size was monitored two to three times per week until the end point. When tumours were palpable, mice were randomized to treatment of sorafenib (12.5 mg/kg three times per week) or vehicle (saline) through intraperitoneal injection. To minimize toxicity, we delivered an established low dose of sorafenib. Tumour size was monitored two to three times per week until the end point.

### Statistical analysis

All animal experiments and in vitro assays were repeated at least two independent experiments. All data are shown as mean ± SEM. Statistical analyses were performed with GraphPad Prism software v.9 using one or two-way analysis of variance (ANOVA) followed by Tukey’s test/Tukey’s multiple comparison test, or Microsoft Excel 2019 using unpaired two-tailed Student’s *t*-test as described in figure legends. In order to apply the *t*-test, the Kolmogorov–Smirnov Test (n > 50) or Shapiro–Wilk test was used to assess the normal distribution of data, whose equal variance was measured by the Bartlett’s test. Kaplan–Meier analysis was used for survival analysis, and the log rank test was used to estimate the difference in survival probability. The Cox proportional hazards model was used to identify independent factors affecting survival. *P* < 0.05 was considered statistically significant.

## Results

### Dietary methionine promotes HCC by proteome remodeling

**Fig. 1:**
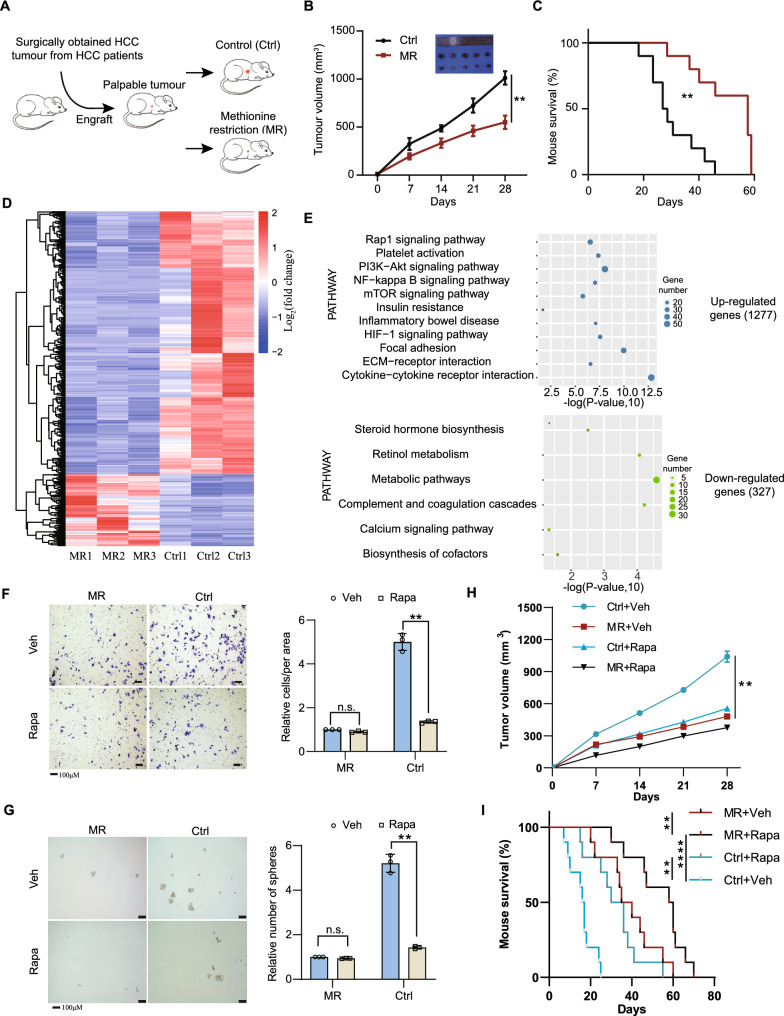
The effect of dietary methionine restriction on mTOR signaling and cancer progression. **A** Schematic of experimental design using HCC PDX model. Treatment, n = 5 mice per group. **B** Tumour growth curves and images of tumours at the end point (Day 28). **C** Survival analysis of mice under Ctrl or MR conditions. **D** Heatmap displaying global transcriptional changes of liver tissues between Ctrl and MR described in **A**. Red color represents the genes’ expression upregulated, while blue means the genes’ expression decreased. **E** KEGG pathway enriched for up-regulated and down regulated genes of liver tissues by comparing Ctrl to MR conditions. **F** Migration of SNU449 cells (left panel) with quantification data on the right panel. **G** Sphere formation efficiency of SNU449 cells (left panel) with quantification (right panel). **H**,** I** Tumour volumes and survival analysis of mouse allografts. n = 5 mice per group. Data are means ± SEM. Group differences were analyzed by two-tailed Student’s t test (**B**, **C**), two-way ANOVA followed by Tukey’s multiple comparison test (**F**, **G**, **H**) or log-rank test (**I**) (**p < 0.01, ****p < 0.0001). n.s., not significant. Ctrl, Control and MR, Methionine restriction

Dietary methionine has a remarkable effect on cancer progression [31, 32], but its effect on the HCC progression remains further explored. We first considered patient-derived xenograft (PDX) models of liver cancer for 2 weeks, then mice were randomized into two groups when the tumour was palpable, with one group fed a standard diet (0.86% methionine, w/w) and the other fed a methionine-restricted diet (0.12% methionine, w/w) (Fig. 1A). Methionine restriction (MR) significantly inhibited tumour growth and prolonged mice lives (Fig. 1B, C), which was also observed in another disease model induced by N-nitrosodiethylamine (DEN) and carbon tetrachloride (CCl_4_) (Additional file 1: Fig. S1A–C). Note that the inhibitory effect was not because of caloric restriction as similar amounts of food intake were observed for both groups.

This intriguing phenomenon promoted us to study what might cause this striking difference during methionine restriction compared to the control set. Therefore, we determined the consequences of MR on gene expression using RNA sequencing (RNA-seq) in the liver. As shown in Fig. 1D, MR greatly remodeled the gene expression in the liver. In the control set, 327 genes out of 30,058 detected genes were downregulated. These genes were involved in steroid hormone biosynthesis, retinol metabolism, and other metabolic processes, suggesting that MR could stimulate specific metabolic pathways (Fig. 1E). Interestingly, 1277 genes were significantly upregulated in the control diet (p-value < 0.05, fold change > 2) (Fig. 1D and Additional file 2: Table S1A). These genes are mainly involved in the cytokine-cytokine receptor interaction, inflammatory bowel disease and several signaling pathways, including mTOR signaling pathway. The elevated expression of canonical mTOR target genes, including c-Myc, fzd9, prkcb and wnt9b, was confirmed by reverse transcription-quantitative PCR (qRT-PCR) (Additional file 1: Fig. S1D). The upregulation of protein c-Myc was further confirmed by western blotting (Additional file 1: Fig. S1E). We also found that SIRT4 downregulation, TRIM32 upregulation and unchanged level of MAT2A (Additional file 1: Fig. S1E) in the Ctrl liver samples compared to that in MR. These results consistent with the prevailing idea that mTORC1 is a central hub of nutrient signaling to coordinate cellular metabolism and growth in response to nutrient availability [13, 33] (Fig. 1E). We hypothesized that mTORC1 might play a role in this striking difference between dietary restriction and the control set. Indeed, immunohistochemical staining revealed that the mTORC1 signaling was activated in the control set, as indicated by higher phosphorylation level of p70 S6 Kinase, a putative mTORC1 substrate (Additional file 1: Fig. S1F). To further confirm our hypothesis, we sought to investigate the effect of rapamycin, an inhibitor of mTORC1, on the HCC development. Rapamycin intensively diminished the cellular growth rate, migration, regeneration and tumorigenesis compared to the control set, and the effect was further augmented by methionine restriction (Fig. 1F–I and Additional file 1: Fig. S1G, H). These results indicated the essential role of mTORC1 in initiating HCC tumorigenesis in response to the availability of methionine.

### mTOR signaling is activated by dietary methionine for the HCC progression

**Fig. 2:**
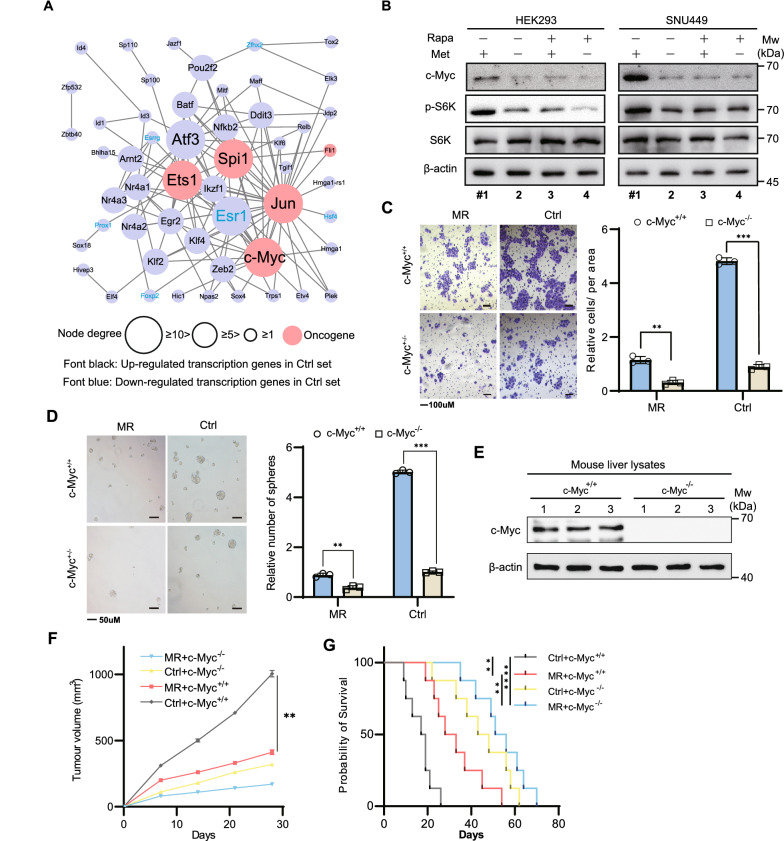
mTORC1 activates c-Myc to promote HCC tumorigenesis. **A** String interaction of differentially expressed transcription factors upon methionine restriction. **B** Western blot analysis of indicated proteins in the presence or absence of rapamycin (20 nM) when HEK293 or SNU449 cells were grown in Medium with or without methionine. **C, D** Migration abilities of primary liver cells derived from c-Myc^+/+^ and c-Myc^−/−^ mice under control or methionine restriction conditions. Quantification was shown in the right panel. **D** Sphere formation efficiency of primary liver cells derived from c-Myc^+/+^ and c-Myc^−/−^ mice under control or methionine restriction conditions. Quantification was displayed in the right panel. **E** Immunoblot showing c-Myc protein level in the liver lysates of c-Myc^+/+^ and c-Myc^−/−^ mice. **F**,** G** Tumour volumes and survival analysis of c-Myc^+/+^ and c-Myc^−/−^ mice. n = 6 mice per group. Data are means ± SEM. Group differences were analyzed by two-way ANOVA followed by Tukey’s multiple comparison test (**C**, **D**, **F**) or log-rank test (**G**) (**p < 0.01, ****p < 0.0001). n.s., not significant

Next, we would like to investigate how the cancer cells alter gene expression to maintain fast proliferation rate after tumour initiation by mTORC1 signaling. Since transcription factors are proteins involved in the process of converting DNA to RNA, we found that 61 transcription factors were upregulated while only 18 were downregulated (Additional file 2: Table S1B). c-Myc, Jun, Spi1, Ets1, Atf3 and Esr1 plays central hubs in the transcription networks with the first 4 proteins being recognized as oncogenes in the Uniprot database (Fig. 2A). Interestingly, only c-Myc acts downstream of mTORC1 signaling [15], therefore, we explored whether the mTORC1 signaling is responsible for the robust c-Myc expression in a methionine dependent manner. Methionine withdraw resulted in a strong reduction in c-Myc expression and the phosphorylation level of p70 S6 Kinase in HEK293 cells, a signal indicative of mTORC1 inhibition (Fig. 2B and Additional file 1: Fig. S2A). However, rapamycin treatment remained the c-Myc expression at low level despite of increased methionine. We also observed similar results in the SNU449 cells (Fig. 2B and Additional file 1: Fig. S2A). These results together demonstrate that c-Myc expression is dependent on mTORC1 signaling in response to methionine availability. The activation of oncogenic gene c-Myc by mTORC1 may facilitate c-Myc mediated transcription, thereby providing metabolic demanding for the cancer progression.

**Fig. 3:**
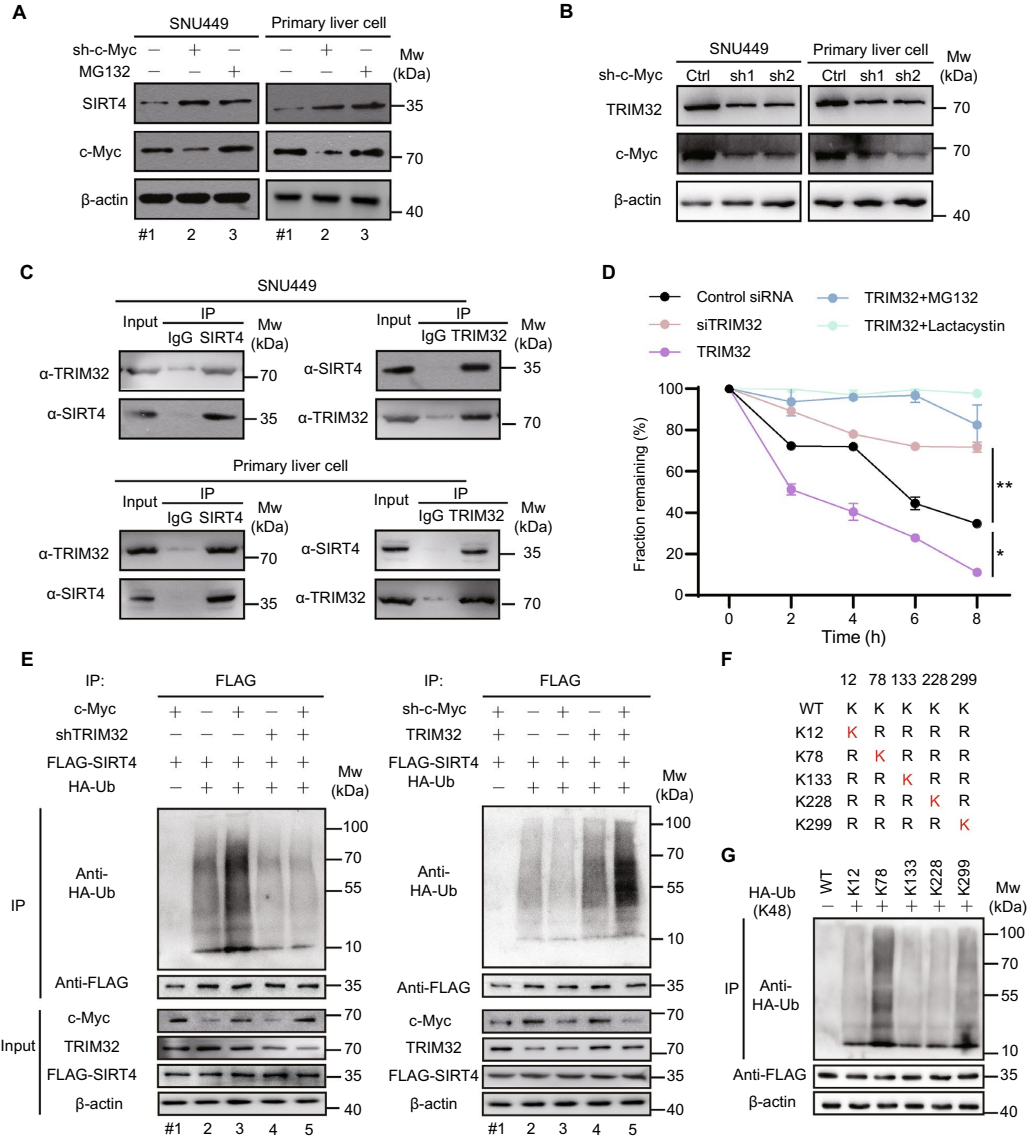
c-Myc promotes TRIM32 mediated proteasomal degradation of SIRT4. **A** Western blot analysis of SIRT4 and c-Myc expression in the whole cellular lysates treated with c-Myc shRNA or MG132 for 48 h. **B** Western blot analysis of TRIM32 and c-Myc expression in whole cellular lysates treated with c-Myc shRNA. **C** SIRT4 interacts with TRIM32 in vivo. SNU449/HEK293 cell lysates were immunoprecipitated (IP) with control IgG, anti-SIRT4 or anti-TRIM32 antibodies, and then the precipitated proteins were detected by anti-TRIM32 or anti-SIRT4 antibodies, respectively. **D** SIRT4 degradation curves under various conditions. TRIM32 was knockdown with shRNA or forced overexpressed in SNU449 cells with or without being treated by proteasome inhibitors MG132 or lactacystin. After being treated with protein synthesis inhibitor cycloheximide (CHX; 50 μg/ml), the above cells were collected at the indicated hours. SIRT4 protein levels were detected by immunoblotting in the SNU449 total cell lysates. **E** SNU449 cells expressing FLAG-SIRT4 and co-expressing with or without hemagglutinin-tagged ubiquitin (HA-Ub) were co-transfected c-Myc and TRIM32 shRNA (left) or co-transfected c-Myc shRNA and TRIM32 (right) for 48 h. After being treated with 5 µM MG132 for 8 h, cells were collected and IP was performed by using FLAG antibody. Polyubiquitination of FLAG-SIRT4 were detected by anti HA-Ub antibody. **F** The indicated lysine sites of SIRT4 were mutated into arginine. **G** Lysates from SNU449 cells expressing the above SITR4 site mutants and co-expressing with or without hemagglutinin-tagged ubiquitin (HA-Ub) were pulled down with anti-FLAG, and then immunoblotted with anti HA-Ub antibody. Group differences were analyzed by two-tailed Student’s t test (**D**) (*p < 0.05, **p < 0.01)

To explore the role of c-Myc in the HCC tumorigenicity during methionine restriction, we then expressed doxycycline (DOX) inducible c-Myc shRNA in primary liver cells (Additional file 1: Fig. S2B, C). The inducible knockdown reduced the proliferation rate of these cells in the standard diet to a similar extent as that of methionine restriction (Additional file 1: Fig. S2D). We also inoculated these cells into nude mice. When tumours reached ~ 65 mm^3^ in volume, experimental mice groups were subjected to DOX with or without MR in the diet. The induction of c-Myc silencing by DOX substantially attenuated the growth of the tumours, almost similar to the MR set (Additional file 1: Fig. S2E). Indeed, c-Myc ablation can significantly reduce migration and sphere formation abilities of HCC cells (Fig. 3D, E), and reverse DEN-induced HCC tumorigenesis and prolonged the mouse lives (Fig. 3F, G). These results together indicated that mTORC1 signaling promotes HCC tumorigenesis through c-Myc activation.

### c-Myc promotes TRIM32-mediated proteasomal degradation of SIRT4

Our previous finding revealed that SIRT4 ablation activated mTOR activity and increased its downstream gene c-Myc while SIRT4 expression altered mTOR phosphorylation and c-Myc gene expression in an opposite way [23]. However, the vice verse effect has not been investigated yet. Compared to that of the MR set, the lower level of SIRT4 in the control set (Additional file 1: Fig. S3A, B) also indicated a possible link between mTORC1-c-Myc pathway and SIRT4 in the HCC tumorigenesis.

We found that SIRT4 at the transcriptome remained almost unchanged (Additional file 2: Table S1A). Therefore, we deliberated whether c-Myc might play a role in repressing SIRT4 expression at the protein level. Interestingly, knockdown of c-Myc significantly increased SIRT4 expression, and the suppressive effect of c-Myc on SIRT4 expression was attenuated by treating the cells with proteome inhibitor MG132 (Fig. 3A and Additional file 1: Fig. S4A). These results implied that c-Myc promoted proteasome-mediated SIRT4 degradation, most probably through regulating E3 ligase gene expression. To identify the potential E3 ligase that might be responsible for SIRT4 turnover, we analyzed a known SIRT4 interactome [22]. We found that TRIM32 as a putative candidate (Additional file 1: Fig. S4B), as c-Myc knockdown in different cell lines reduced TRIM32 expression level (Fig. 3B and Additional file 1: Fig. S4C). Western-blot analysis confirmed the interaction between SIRT4 and TRIM32 (Fig. 3C).

Cycloheximide (CHX) chase assays further revealed the positive role of TRIM32 in SIRT4 stability. Forced overexpression of TRIM32 accelerated the degradation rate of SIRT4, while TRIM32 knockdown strongly stabilized SIRT4, however, the effect of TRIM32 on SIRT4 degradation was abolished by treating the cells with proteasome inhibitor MG132 or lactacystin (Fig. 3D and Additional file 1: Fig. S4D). To assess whether TRIM32 ubiquitinates SIRT4, we purified SIRT4 in SNU449 cells that were co-transfected with FLAG-SIRT4, hemagglutinin-tagged ubiquitin (HA-Ub), c-Myc/TRIM32 shRNA (left) or c-Myc shRNA/TRIM32 (right) (Fig. 3E). Western blot analysis of SIRT4 immunoprecipitation showed that c-Myc overexpression enhanced ubiquitination of SIRT4, which was attenuated by TRIM32 knockdown (Fig. 3E, left panel and Additional file 1: Fig. S4E). In contrast, TRIM32 overexpression blunted the inhibitory effect of c-Myc silence on SIRT4 ubiquitination (Fig. 3E, right panel and Additional file 1: Fig. S4F). Point mutation of SIRT4 (lysine to arginine) showed that ubiquitination sites of SIRT4 are in the lysines 78 and 229, with the former as the main site (Fig. 3F, G and Additional file 1: Fig. S4G). Moreover, we did co-immunoprecipitation of TRIM32 in cells where truncated SIRT4 with different length was expressed. WB analysis showed that TRIM32 was strongly bound to SIRT4 with length 1–314 or 45–314, whereas TRIM32 interacted weakly to SIRT4 with length 100–314 or 150–314 (Additional file 1: Fig. S5A). Indeed, SIRT4 K78R mutant reduced association with TRIM32 while SIRT4 K299R mutant remained bound to TRIM32 (Additional file 1: Fig. S5B). Furthermore, TRIM32 overexpression had less effect on the expression of SIRT4 K78R mutant than that of SIRT4 K299R mutant (Additional file 1: Fig. S5C). As lysine 78 is located at NAD^+^ binding region, SIRT4 K78R mutant also reduced cell proliferation rate (Additional file 1: Fig. S5D). Taken together, these results provide convincing evidences that c-Myc promotes SIRT4 degradation by mediating the expression of the ubiquitinase TRIM32.

### SIRT4 attenuates c-Myc-promoted tumorigenesis by altering methionine metabolism

Given that c-Myc negatively regulates SIRT4 and SIRT4 has been reported as a cancer suppressor [23], we next explored whether there is any interplay between c-Myc and SIRT4 for the cancer progression. Interestingly, SIRT4 overexpression strikingly suppressed c-Myc induced cell proliferation and stemness (Additional file 1: Fig. S6A-C), a strong hint that SIRT4 retarded c-Myc mediated tumorigenesis through an unknown mechanism.

**Fig. 4:**
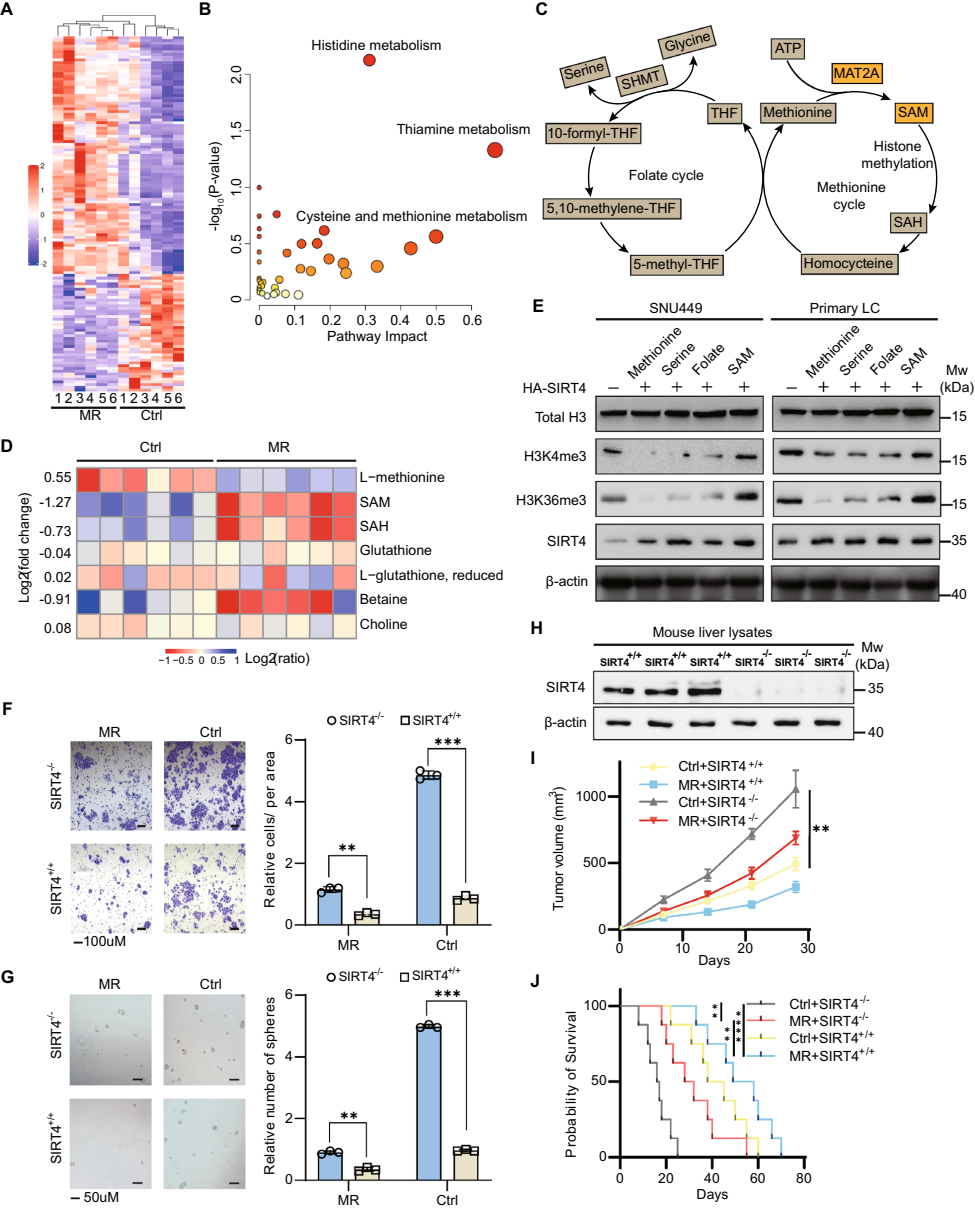
Effect of SIRT4 overexpression on methionine cycle and HCC tumorigenesis. **A** Heatmap displaying metabolite changes upon methionine restriction (MR). The red color indicates detected metabolites were increased while the blue ones decreased between MR and Ctrl conditions in six biological replicates. **B** Pathway enrichment analysis of differentially detected metabolites (fold change > 1.5, p-value < 0.05) upon methionine restriction in MetaboAnalyst 4.0. The circle color is indicative of the level of enrichment significance, with yellow being low and red being high. The circle size is proportional to the pathway impact value. **C** Schematic of the methionine cycle. **D** The changes of specific metabolites upon methionine restriction. Blue means increased while red indicates decreased. **E** Western blot analysis of modified histones in the cell culture supplemented with specific metabolites. Total histone H3 was used as a loading control. **F**, **G** Migration and sphere formation efficiency of primary liver cells derived from SIRT4^+/+^ and SIRT4^−/−^ mice under control or methionine restriction conditions. Quantification was shown in the right panel. **H** Immunoblot showing SIRT4 protein level in the liver lysates of SIRT4^+/+^ and SIRT4^−/−^ mice. **I**,** J** Tumour volumes and survival analysis of SIRT4^+/+^ and SIRT4^−/−^ mice. n = 5 mice per group. Data are means ± SEM. Group differences were analyzed by two-way ANOVA followed by Tukey’s multiple comparison test (**F**, **G**, **I**) or log-rank test (**J**) (**p < 0.01, ***p < 0.001 ****p < 0.0001). n.s., not significant

To dissect the possible role of MR in the metabolism, we analyzed the metabolites changes upon methionine restriction in vivo (Fig. 4A). MR profoundly rewired the metabolism in mouse liver. Of 744 metabolites identified by liquid chromatography-tandem mass spectrometry (LC–MS/MS), 51 metabolites were significantly increased and 243 were decreased (p < 0.05) (Additional file 3: Table S2), with pathways enriched in histidine metabolism, thiamine metabolism and cysteine and methionine metabolism (Fig. 4A, B). We checked the metabolites related with methionine metabolism and found that SAM and S-adenosyl-homocysteine (SAH) declined in the MR set (Fig. 4C, D). To explore the possible relationship between SIRT4 and methionine metabolism, we measured the relative levels of metabolites related with methionine metabolism by comparing SIRT4 overexpression to the control cells. To our surprise, SAM, SAH, homocysteine from methionine metabolism was greatly reduced while other one-carbon cycle related metabolites remained almost unchanged in the SIRT4 overexpression cells (Additional file 1: Fig. S7A). Indeed, SIRT4 overexpression strongly reduced histone trimethylation (Additional file 1: Fig. S7B,C), which has important roles in defining chromatin states, most notably at active genes. Histone methylation are regulated by balanced metabolite levels in the one-carbon and methionine metabolism, for example, methionine, serine and folate [34]. These metabolites contribute epigenetic regulation by providing one-carbon units. Interestingly, only SAM supplementation resorted the level of histone methylation (Fig. 4E and Additional file 1: Fig. S7D).

These results above indicate that SIRT4 plays a key role in the methionine metabolism and inhibits SAM production. Therefore, it was of interest to examine the role of SIRT4 in the HCC in vitro and in vivo. SIRT4 deletion dramatically increased migration, and sphere formation abilities of primary liver cells, and the effect was more significant in the control set (Fig. 4F, G). Consistent with in vitro observations, SIRT4 ablation in mice displayed a more remarkable effect in the promotion of tumour formation in the control set and significantly decreased the life of these mice (Fig. 4I-J). In contrast, SIRT4 overexpression with combination of MR treatment greatly reduced the cell proliferation, migration, sphere formation abilities and tumour size, and prolonged mouse survival (Additional file 1: Fig. S8A-F). Moreover, mice of inducible SIRT4 depletion in the control set showed strikingly increased tumorigenesis and shortened the mice’s lives (Additional file 1: Fig. S8G-I). Collectively, these findings demonstrated that SIRT4 suppress HCC progression by regulating methionine metabolism.

### SIRT4 mediates mono-ADP-ribosylation of MAT2A

**Fig. 5:**
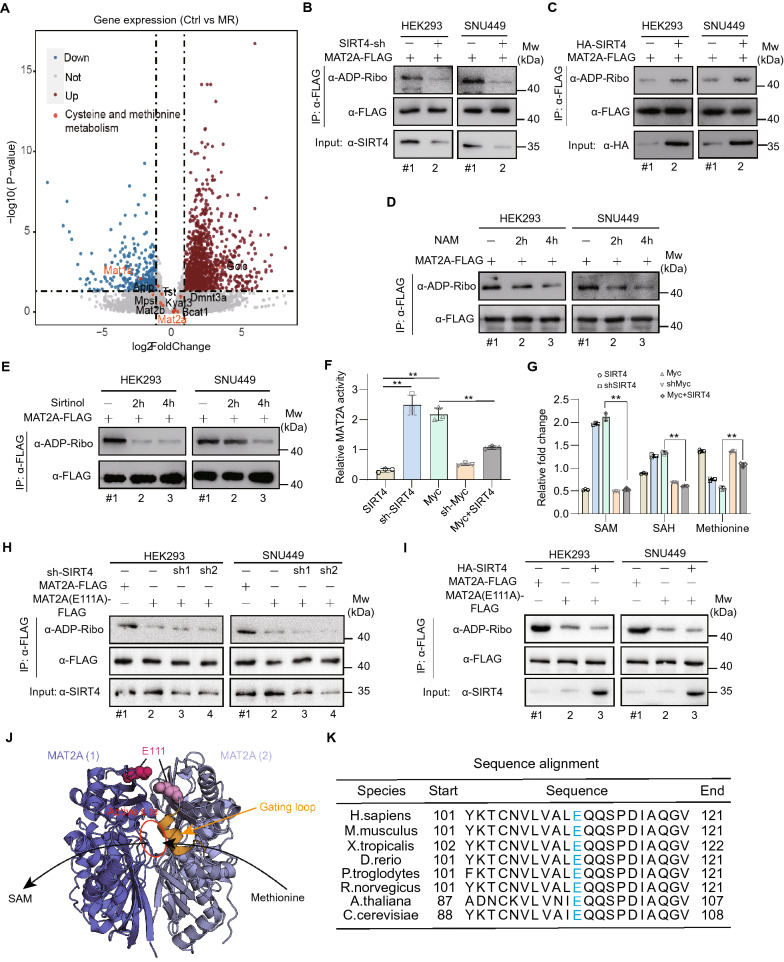
SIRT4 regulates methionine metabolism through mediating MAT2A ADP ribosylation. **A** Volcano plot showing differential gene expression in mice’s livers upon methionine restriction. Red circles indicates genes increased in the Ctrl set while blue ones means genes decreased in the MR set. **B** Western blot analysis of MAT2A mono-ADP-ribosylation (MARylation) in HEK293 and SNU449 cells in which SIRT4 was silenced (shRNA). **C** The MARylation of MAT2A was measured by western blot in HEK293 or SNU449 cells stably expressing HA tagged SIRT4. **D** Western blot analysis of MAT2A MARylation in the FLAG immunoprecipitated samples from HEK293 or SNU449 cells treated with nicotinamide (NAM) for indicated times. **E** Western blot analysis of MAT2A MARylation in the FLAG immunoprecipitated samples from HEK293 or SNU449 cells treated with Sirtinol for indicated times. **F** Relative activity of MAT2A was shown in the SNU449 cells in which either c-Myc or SIRT4 was knockdown or overexpressed or c-Myc and SIRT4 overexpressed together. **G** Abundances of intracellular primary methionine cycle metabolites were compared between the control cells and the SNU449 cells where either c-Myc or SIRT4 was knockdown or stably overexpressed or c-Myc and SIRT4 overexpressed together. **H** MARylation level of mutant FLAG-MAT2A-E111A ectopically expressed in MAT2A-knockout HEK293 or SNU449 cells was measured by using anti-ADP ribosylation antibody when SIRT4 was silenced. **I** MARylation level of mutant FLAG-MAT2A-E111A ectopically expressed in MAT2A-knockout HEK293 or SNU449 cells was measured by using anti-ADP ribosylation antibody when SIRT4 was overexpressed. **J** MAT2A dimer are shown in blue or lightblue (PDB ID: 4NDN). The active site was shown in red circle. **K** E111 in MAT2A is conserved. Protein sequence alignment surrounding E111 colored in light blue from indicated species. Data are means ± SEM. Group differences were analyzed by two-tailed Student’s multiple comparison test (**F**, **G**) (**p < 0.01)

We further sought to investigate how SIRT4 regulates methionine metabolism. We first checked the transcriptome changes of genes related with methionine metabolism, and found that MAT1A significantly decreased while MAT2A and MAT2B remained almost unchanged (Fig. 5A and Additional file 1: Table S1A). Therefore, we speculated that SIRT4 might modulate the methionine metabolism by regulating post-translational modifications of MAT2A/MAT2B. As reported in published studies, SIRT4 potentially interacted with MAT2A [21, 22]. Western blot analysis confirmed that SIRT4 was physically associated with MAT2A (Additional file 1: Fig. S9A), which is a mono-ADP-ribosylation protein (MARylation) [35]. To characterize the role of SIRT4 as an ADP-ribosyltransferase, we investigated whether altered expressed SIRT4 affect the MARylation level of MAT2A. Specifically, SIRT4 knockdown markedly reduced MARylation level of MAT2A (Fig. 5B and Additional file 1: Fig. S9B), while SIRT4 overexpression strongly increased MAT2A MARylation (Fig. 5C and Additional file 1: Fig. S9C). ADP-ribosyltransferase activity is inhibited by two structurally distinct inhibitors of sirtuins, nicotinamide (NAM) and sirtinol [36]. Inhibition of SIRT4 activity by either NAM or sirtinol significantly deceased MARylation level of MAT2A (Fig. 5D, E and Additional file 1: Fig. S9D, E), further validating that MAT2A MARylation is catalyzed by SIRT4.

c-Myc negatively regulates SIRT4 expression. Indeed, c-Myc overexpression reduced the MARylation on MAT2A, while SIRT4 overexpression attenuated the inhibitory effect of c-Myc on MAT2A MARylation in different cell lines (Additional file 1: Fig. S9F, G). Furthermore, knockdown of c-Myc and SIRT4 alone or together had the vice versa effect (Additional file 1: Fig. S9F, G). Consistently, forced overexpression of c-Myc increased MAT2A activity, whereas SIRT4 overexpression suppressed the promoting effect of c-Myc on MAT2A activity (Fig. 5F). As a consequence, SIRT4 overexpression reduced SAM production (Fig. 5G). In contrast, c-Myc overexpression promoted elevation of SAM, while c-Myc knockdown reduced SAM level. As expected, SIRT4 overexpression diminished the promoting effect of c-Myc on SAM level (Fig. 5G). Taken together, these data demonstrate that c-Myc relieves MAT2A MARylation by promoting TRIM32 mediated SIRT4 degradation, which consequently enhances the role of MAT2A in the methionine metabolism.

We mutated each of eight putative MARylation sites individually into alanine (A) (Additional file 1: Fig. S10A) [37] and examined MARylation level. Mutation of glutamic acid 111 (E111A), but not other glutamic acid or aspartic acid residues to alanine, lead to a remarkable reduction in MAT2A MARylation (Additional file 1: Fig. S10B). Either SIRT4 knockdown or overexpression did not affect MARylation level of E111A mutant (Fig. 5H, I and Additional file 1: Fig. S10C, D). In addition, NAM or sirtinol treatment had almost no effect on the MARylation level and activity of MAT2A-E111A mutant (Additional file 1: Fig. S10E, F). These results indicate that E111 is a major MARylation site in MAT2A. Importantly, E111 is located in the substrate binding region and is in the vicinity of the gating-loop residue Q113, which directly contacts methionine and is proposed to play a critical role in positioning methionine for catalysis (Fig. 5J) [38, 39]. The modified ADP moiety is larger than methionine, thus, MARylation on E111 may prevent methionine from accessing the gating-loop residue Q113, resulting in low catalytic efficiency. Note that E111 is evolutionarily conserved from Saccharomyces cerevisiae to mammals (Fig. 5K).

MAT2A contributes to tumour progression in multiple types of cancers by promoting methionine metabolism [26, 27, 40–42]. Interestingly, SIRT4 and MAT2A co-expression leads to marked decrease in the cell proliferation (Additional file 1: Fig. S10G). In contrast, this effect was not observed in the MAT2A-E111A mutant cell line, as it abrogated SIRT4-induced suppression effect on cell proliferation (Additional file 1: Fig. S10G). Consistently, the tumour growth was strongly inhibited by SIRT4 overexpression but increased in the mice injected with MAT2A-E111A mutant (Additional file 1: Fig. S10H, I). Cellular treatment with FIDAS-5, an inhibitor of MAT2A [3], strongly affected MAT2A activity and its related histone methylation (Additional file 1: Fig. S11A-C), as well as cell proliferation and tumour growth (Additional file 1: Fig. S11D, E), similar to that observed in cells stably overexpressing SIRT4 (Additional file 1: Fig. S10G, H). These results demonstrate that SIRT4 exerts its role in the methionine metabolism through MARylation on MAT2A, thereby inhibiting cellular proliferation and tumour growth.

### SIRT4 affects transcription through regulating methionine metabolism

Methionine is a key metabolic dependence for the tumour initiation and progression, as methionine metabolism determines levels of histone methylation by modulating SAM and SAH, which influences genomic architecture and subsequent gene expression [3, 31]. Therefore, we took an oncogenic transcription factor c-Myc as an example to investigate how SIRT4 affects gene transcription by modulating methionine metabolism.

**Fig. 6:**
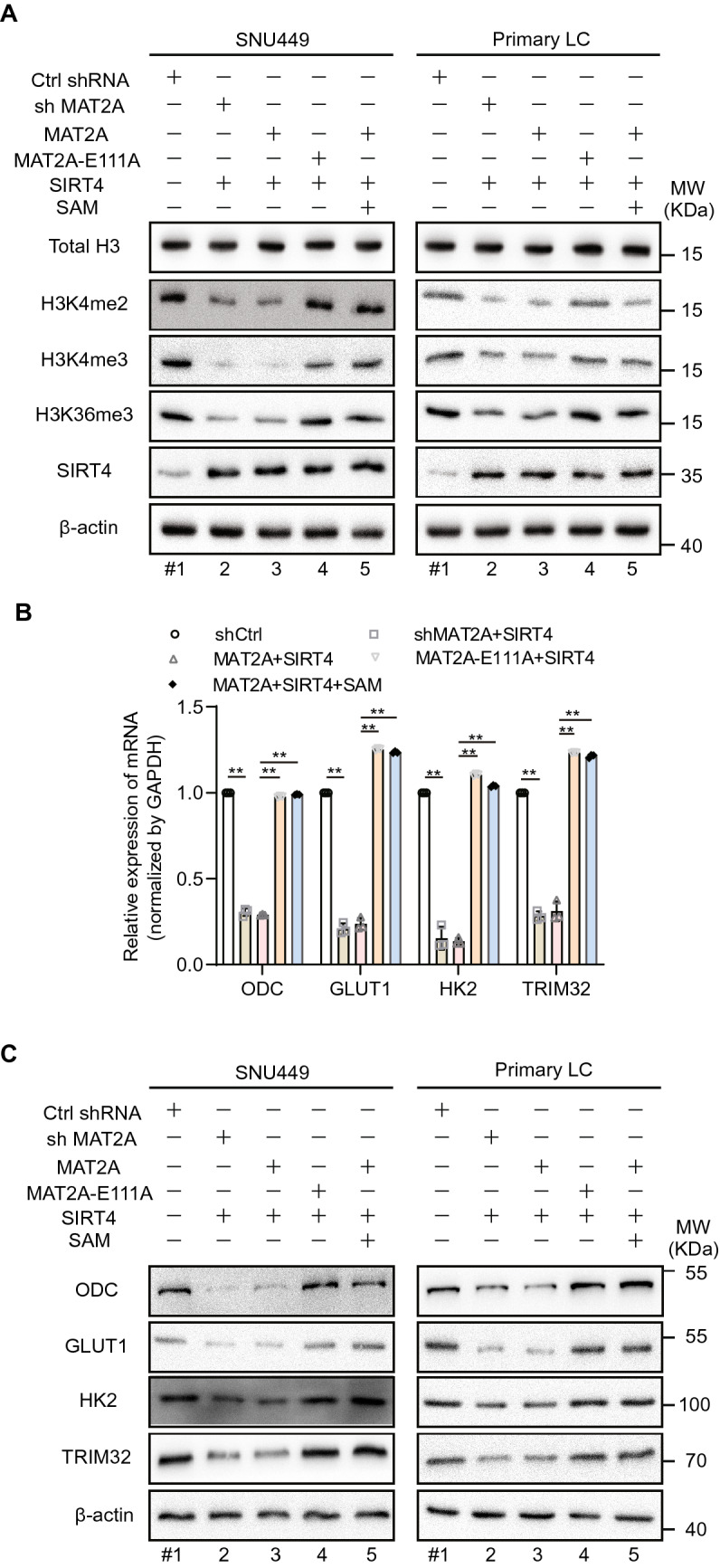
SIRT4 modulates gene expression by regulating methionine metabolism. **A** Western blot analysis of histone methylation level. Histone H3 was used as a loading control. **B** The mRNA levels of indicated genes were determined by quantitative real-time PCR (qRT-PCR) in the SNU449 cells. **C** The indicated proteins were assessed by western blot in the SNU449 or primary liver cells. Data are means ± SEM. Group differences were analyzed by two-way ANOVA followed by Tukey’s multiple comparison test (**B**, **D**) (**p < 0.01)

SIRT4 overexpression greatly reduced levels of H3K4me3, H3K4me2 and H3K36me3, but not in the MAT2A-E111A mutant cells (Fig. 6A and Additional file 1: Fig. S12A). These results demonstrate that SIRT4 has an indirect effect on the histone methylation by inhibiting methionine metabolism through MAT2A MARylation. Then we checked the epigenetic change at c-Myc gene loci. Indeed, SIRT4 overexpression decreased H3K4me3 and H3K36me3 at the c-Myc promoter, while SIRT4 loss increased H3K4me3 and H3K36me3 at the c-Myc promoter (Additional file 1: Fig. S12B, C). SIRT4 overexpression also altered repressive histone methylation marks, in case of H3K9me2, H3K9me3, and H3K27me3 (Additional file 1: Fig. S12D). To further analyze the consequence of methionine rewiring on gene expression, we specifically checked c-Myc-targeted genes in MAT2A, MAT2A-E111A mutant and SIRT4 expressing SNU449 cells that were supplemented with or without SAM [43]. These genes play keys roles in the cancer metabolism, for example, ODC in the polyamine synthesis and HK2 in the glycolysis. Either MAT2A knockdown or SIRT4/MAT2A co-expression decreased the expression of c-Myc-targeted genes (Fig. 6B). However, SAM supplementation restored these genes’ expression (Fig. 6B), showing simultaneous recovery of their mRNA as well as histone methylation. Interestingly, SIRT4 did not have obvious effect in the MAT2A-E111A mutant cells (Fig. 6B). These results were further confirmed at the protein level (Fig. 6C and Additional file 1: Fig. S12E). These results all together show that SIRT4 has a key regulatory role in methionine metabolism to modulate gene expression.

### SIRT4 sensitizes HCC to targeted therapy with Sorafenib

**Fig. 7:**
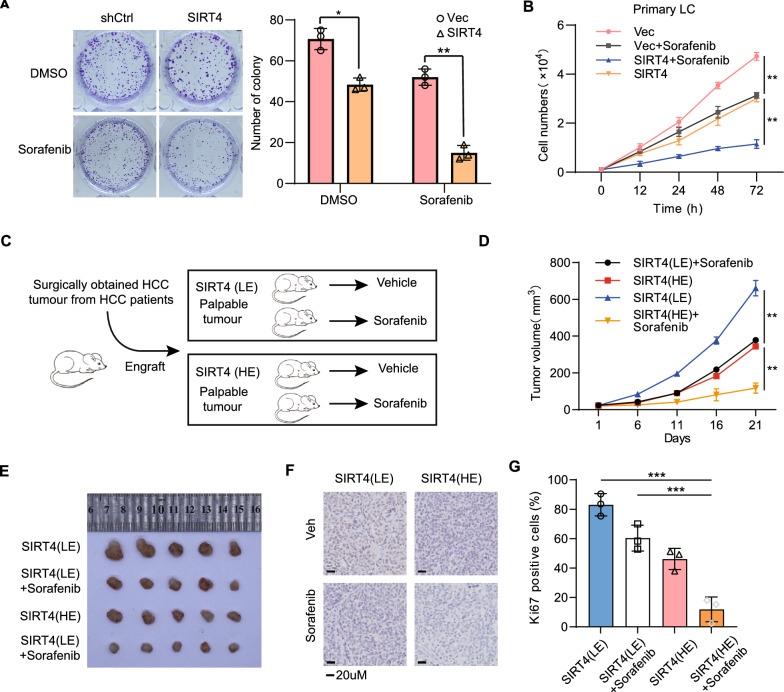
SIRT4 increases the sensitivity of HCC cells to chemotherapeutic drug Sorafenib. **A** Colony formation of primary HCC cells expressing shCtrl or SIRT4 in the absence or presence of Sorafenib. **B** Growth curves of primary HCC cells expressing shCtrl or SIRT4 treated with or without Sorafenib. **C** Experimental design using HCC PDXs, where mice were injected with human HCC cells with SIRT4 low expression (LE) or high expression (HE). **D**,** E** Tumour volume curves and images at the end the point. **F**,** G** IHC analysis and quantification of Ki67-positive cells of PDX described in **C**. n = 5 mice per group. Group differences were analyzed by two-way ANOVA followed by Tukey’s multiple comparison test (**A**, **B**, **D**, **G**) (*p < 0.05, **p < 0.01, ***p < 0.001)

Sorafenib, a first-line dual-target inhibitor, targets both the serine-threonine kinase Raf and the tyrosine kinases VEGFR/PDGFR. However, its effectiveness for the treatment of many advanced-stage HCC patients is hampered by drug resistance due to HCC heterogeneity [44]. To evaluate the potential of methionine metabolism as a therapeutic target in HCC, we explored whether SIRT4 could synergize sorafenib in in-vitro and in-vivo. Strikingly, high expression of SIRT4 synergized with sorafenib treatment, leading to more remarkable inhibition of cell proliferation and colony formation (Fig. 7A, B). In line with this, similar effects were observed in the suppression of tumor growth (Fig. 7C–G). Fold changes of metabolites, involved in nucleotide metabolism and redox balance, were also highly related to both the mechanistic action of sorafenib and SIRT4(Additional file 1: Fig. S13A). Next, we supplemented methionine metabolism related nutrients to primary liver cells with or without high expression of SIRT4 in the presence of sorafenib. SAM, along with other supplements, only partially alleviated the inhibition of cell proliferation due to SIRT4 overexpression/sorafenib (Additional file 1: Fig. S13B, C). Inhibition of methionine metabolism by SIRT4 synergizes with sorafenib, thereby retarding the growth of HCC tumour by disrupting nucleotide metabolism and redox balance.

### Clinical significance of SIRT4 mediated ADP ribosylation

**Fig. 8:**
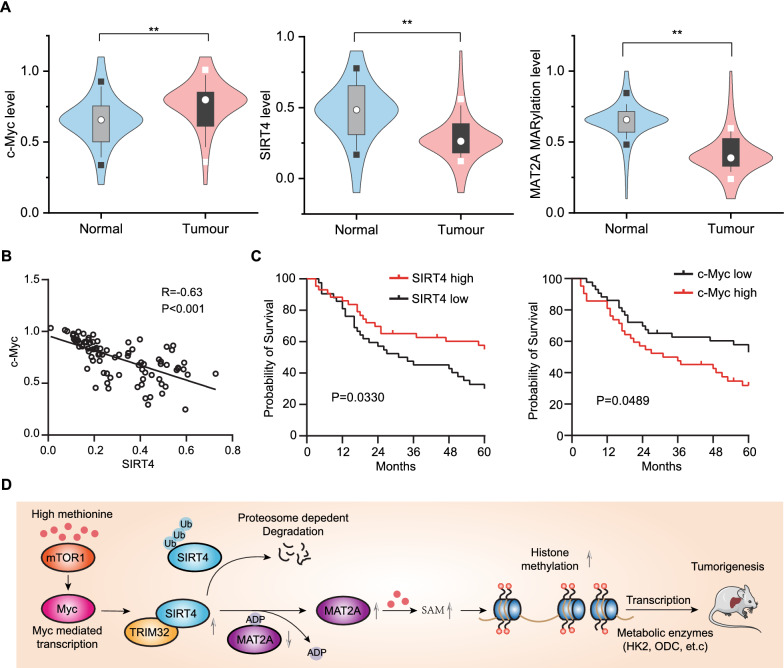
Myc promoted SIRT4 degradation to reduce ADP ribosylation of MAT2A in the HCC. **A** Protein levels of c-Myc, MAT2A and MARylation level of MAT2A were determined by western blot from 85 pairs of tumour tissues. In the inserted boxplots, the circles indicate the median; the square indicate the 5th–95th percentiles. The statistical analysis was performed by the two tailed Student’s t-test (**p < 0.01). **B** Correlation between Myc and SIRT4 in HCC patients. 85 tissue pairs were immunohistochemically stained with indicated antibodies. The pair-wise Pearson correlation coefficient and the corresponding p-value between two genes were calculated. **C** The patients with high SIRT4 expression (n = 43) have longer survival compared to low SIRT4 expression (n = 42). Significance was determined using Kaplan–Meier analysis. **D** Schematic showing that high methionine content is sensed by mTORC1 that triggers c-Myc expression, which promotes TRIM32-mediated SIRT4 degradation. SIRT4 regulates MAT2A activity through MARylation, thereby inhibits MAT2A mediated tumour growth. SIRT4 loss resulted in the low MARylation level of MAT2A, which promotes methionine metabolism to generate SAM. In a feedback, high SAM level triggers transcription of metabolic enzymes to maintain tumour progression. Upward pointing arrows indicate an increase, and downwards pointing arrows indicate a decrease

Our findings above promoted us to determine SIRT4 and c-Myc protein expression levels and ADP ribosylation level of MAT2A in human HCC cancer. We collected 85 pairs of primary HCC tumor samples to perform a direct immunoblotting analysis of the HCC samples (T) and their adjacent normal tissues (N). Of these 85 pair of clinical samples, compared to matched normal liver tissues, a significant increase in the steady level of c-Myc protein but a reduced expression level of SIRT4 in tumour samples (Fig. 8A and Additional file 1: Fig. S14). Accordingly, c-Myc expression level was negatively correlated with SIRT4 expression and MAT2A MARylation (Fig. 8B). Kaplan–Meier analysis revealed that patients with low SIRT4 level tended to have a poorer overall survival compared to patients with high SIRT4 expression (Fig. 8C). Collectively, these data indicate that MAT2A ADP ribosylation is frequently downregulated in HCC tumors that is associated with high c-Myc but with low SIRT4 protein level, which may be potential biomarkers for hepatocellular cancer diagnosis.

## Discussion

Essential amino acid methionine has been implicated in widespread physiological processes and associated in various metabolic benefits, including lifespan extension, obesity prevention and cancer therapy [31]. Methionine metabolism has emerged as a metabolic node in rapidly proliferating cancer cells through modulation of gene expression [3, 32]. Here, we found that mTORC1-cMyc pathway reprograms methionine metabolism and promotes gene expression for the cellular nutrient demanding. As an essential hub in the sensing of nutrient availability, mTOR pathway is frequently upregulated in up to half of HCCs [45]. mTORC1 signaling was activated in response to high content of methionine, which promoted downstream oncogenic protein c-Myc expression (Fig. 8D) [15, 33, 46]. Either mTORC1 inhibition or c-Myc knockout prevented high-content methionine induced HCC tumorigenesis. The dysregulation of c-Myc in cancer cells promoted TRIM32 expression (Fig. 8D), which ubiquitinated SIRT4 for the proteasome-dependent degradation, thereby facilitating methionine cycle by reducing the ADP-ribosylation modification of MAT2A (Fig. 8D). Oncogenic mTORC1-c-Myc pathway has a tight link to methionine metabolism by regulating SIRT4 expression.

As a family of NAD^+^ dependent deacetylases, sirtuins (SIRT1-SIRT7) have life extending effects by regulating cell metabolism through their multiple catalytic functions [16, 17]. They exert complex functions in either promoting or suppressing malignant characteristics in tumours. We found that only SIRT4 specifically interacting with MAT2A closely, stressing the unique role of SIRT4 in regulating methionine metabolism by its ADP transferase activity. Indeed, a new type of post translational modification MARylation strongly regulates MAT2A’s activity. Therefore, MAT2A mediated tumorigenesis was greatly attenuated by SIRT4 overexpression. However, mutation of MAT2A MARylation site blocks the regulatory role of SIRT4 and promotes cancer development. SIRT4 synergizes chemotherapeutic agent Sorafenib for the HCC treatment in mouse, most probably due to the changes of methionine, redox and nucleotide metabolism, as indicated by the mice’s plasma metabolism. These results indicate a possible opportunity to develop therapeutic drugs to disrupt methionine metabolism by targeting MARylation site of MAT2A or stabilizing SIRT4 expression for HCC treatment.

Our study has limitations. Although we have shown mTORC1-c-Myc pathway could strongly regulate the methionine metabolism in the HCC tumorigenesis, methionine metabolism has been extensively shown to be associated to many other metabolic pathways, for example, polyamine biosynthesis, redox biology and folate metabolism. It is of further interests to investigate whether methionine exerts its tumour suppressive role in other unknown mechanism. It is quite possible since many other activated transcription factors were also modulated by the methionine availability, implying that other pathways may interplay with mTORC1-c-Myc pathway in the cancer metabolism reprogramming. Thus, it is tempting to speculate that dynamic gene expression might be coordinated by activation of these transcription factors upon methionine availability. In addition, the relative trade-offs in altering dietary methionine composition needs further studies for its therapeutic use.

## Conclusion

In total, our study uncovers a novel molecular mechanism induced by dietary methionine, which activates mTORC1-c-Myc pathway to reprogram the methionine metabolism by suppressing SIRT4 expression. SIRT4 regulates methionine metabolism through ADP ribosylation modification of MAT2A’s activity, thereby affecting epigenetic regulation of gene expression. This underlying mechanism may enable implementation of dietary interventions for a broad spectrum of cancer therapies.

## Supplementary Information

**Additional file 1.** Additional methods.** Fig. S1.** mTOR senses the availability of methionine.** Fig. S2.** mTORC1 signaling pathway mediated Myc expression to promote HCC tumorigenesis.** Fig. S3.** Negative correlation of MYC and SIRT4 expression.** Fig. S4.** Myc regulates SIRT4 stability through proteasome degradation.** Fig. S5.** Characterization of the interaction between SIRT4 and TRIM32.** Fig. S6.** SIRT4 suppresses Myc promoted HCC tumorigenesis.** Fig. S7.** The effect of SIRT4 on histone methylation through regulating methionine metabolism.** Fig. S8.** The effect of SIRT4 retards cancer progression through regulating methionine metabolism.** Fig. S9.** SIRT4 regulates MARylation of MAT2A.** Fig. S10.** SIRT4 suppresses MAT2A promoted tumorigenesis.** Fig. S11.** The MAT2A inhibitor FIDAS-5 abrogates HCC cell stemness.** Fig. S12.** SIRT4 affects histone methylation and Myc mediated transcription.** Fig. S13.** SIRT4 increases the sensitivity of HCC cells to chemotherapy.** Fig. S14.** Comparison of c-Myc, MAT2A and MARylation level of MAT2A from clinical liver samples.

**Additional file 2: Table S1.** Detected gene levels upon methionine restriction in livers of HCC mouce models.

**Additional file 3: Table S2.** The changes of metabolite levels upon methionine restriction in livers of HCC mouce models.

## Data Availability

The datasets used and/or analyzed during the current study are available from the corresponding author on reasonable request.

## Abbreviations

HCC: Hepatocellular carcinoma
SAM: S-adenosylmethionine
SAH: S-adenosyl-homocysteine
mTORC1: Mammalian target of rapamycin complex 1
ADP: Adenosine diphosphate
DEN: Diethyl-nitrosamine
LC–MS/MS: Liquid Chromatograph-tandem Mass Spectrometry
PDX: Patient-derived xenograft
CCl_4_: Carbon tetrachloride
RNA-seq: RNA sequencing
MR: Methionine restriction
DOX: Doxycycline
CHX: Cycloheximide
HA-Ub: Hemagglutinin-tagged ubiquitin
MARylation: Mono-ADP-ribosylation protein
NAM: Nicotinamide
5-FU: 5-Fluorouracil
