# Supplementary material for: mTORC1-c-Myc pathway rewires methionine metabolism for HCC progression through suppressing SIRT4 mediated ADP ribosylation of MAT2A
Source: Cell Biosci. 2022 Nov 12;12:183. doi: 10.1186/s13578-022-00919-y (PMC9652997; doi:10.1186/s13578-022-00919-y)
Supplement: Supplementary file 1 — Additional file 1. Additional methods. Fig. S1. mTOR senses the availability of methionine. Fig. S2. mTORC1 signaling pathway mediated Myc expression to promote HCC tumorigenesis. Fig. S3. Negative correlation of MYC and SIRT4 expression. Fig. S4. Myc regulates SIRT4 stability through proteasome degradation. Fig. S5. Characterization of the interaction between SIRT4 and TRIM32. Fig. S6. SIRT4 suppresses Myc promoted HCC tumorigenesis. Fig. S7. The effect of SIRT4 on histone methylation through regulating methionine metabolism. Fig. S8. The effect of SIRT4 retards cancer progression through regulating methionine metabolism. Fig. S9. SIRT4 regulates MARylation of MAT2A. Fig. S10. SIRT4 suppresses MAT2A promoted tumorigenesis. Fig. S11. The MAT2A inhibitor FIDAS-5 abrogates HCC cell stemness. Fig. S12. SIRT4 affects histone methylation and Myc mediated transcription. Fig. S13. SIRT4 increases the sensitivity of HCC cells to chemotherapy. Fig. S14. Comparison of c-Myc, MAT2A and MARylation level of MAT2A from clinical liver samples. [file 13578_2022_919_MOESM1_ESM.docx]

**mTORC1-c-Myc pathway rewires methionine metabolism for HCC progression through suppressing SIRT4 mediated ADP ribosylation of MAT2A**

Liang Zhao^1†^, Huizhao Su^2†^, Xiaomeng Liu^1,2†^, Hongquan Wang^1^, Yukuan Feng^1^, Yan Wang^1,2^, Haiqiang Chen^1,2^, Luo Dai^2^, Shihui Lai^1,2^, Siqi Xu^3^, Chong Li^4^, Jihui Hao^1^*, Bo Tang^1^*.

^1^Department of Pancreatic Cancer, Tianjin Medical University Cancer Institute and Hospital, National Clinical Research Center for Cancer, Key Laboratory of Cancer Prevention and Therapy, Tianjin’s Clinical Research Center for Cancer, Tianjin 300060, China.

^2^Department of Hepatobiliary Surgery, The First Affiliated Hospital of Guangxi Medical University, Nanning, 530021, Guangxi, P. R. China

^3^Zhongke Jianlan Medical Research Institute, Beijing, 101400, China

^4^Institute of Biophysics, Chinese Academy of Sciences, Beijing, 100101, China

^†^Liang Zhao, Huizhao Su and Xiaomeng Liu contributed equally to this work.

*Correspondence: Professor Bo Tang, Department of Pancreatic Cancer, Tianjin Medical University Cancer Institute and Hospital, Tianjin 300060, PR China, Email: [tangbo@tmu.edu.cn](mailto:tangbo@tmu.edu.cn) . Professor Jihui Hao, Department of Pancreatic Cancer, Tianjin Medical University Cancer Institute and Hospital, Tianjin 300060, PR China, Email:haojihui@tjmuch.com .

**Additional Methods**

**Coimmunoprecipitation**

Cells were collected in cold PBS and lysed with RIPA buffer (50 mM Tris (pH 7.4), 150 mM NaCl, 1 mM EDTA, 0.1% SDS, 1% Nonidet P-40 (NP-40), 0.5% sodium deoxycholate, 0.5 mM DTT, and protease inhibitor). The lysates were diluted 2- to 4-fold with dilution buffer (50 mM Tris (pH 7.4), 100 mM NaCl, 1 mM EDTA, 0.1% NP-40, 10% glycerol, and protease inhibitor). Then, 2-5 μg of antibodies were added to the diluted cell lysates, and the mixtures were incubated overnight at 4°C. The next day, the protein complexes were isolated with magnetic Protein G Dynabeads for 2 h at 4°C with rotation. The bead–antibody–protein complexes were then washed 4 times with wash buffer (50 mM Tris (pH 7.4), 125 mM NaCl, 1 mM EDTA and 0.1% NP-40) and boiled for Western blot analysis.

**Flow cytometry**

The cells were suspended in 1×10^6^ cells/ml and 5μL Annexin V and Propidium Iodide (PI) staining solution was added to 300μL of the cell suspension. After incubated 10-15 min at room temperature in the dark, stained cells were assayed and quantified using a FACSort Flow Cytometer (BD, San Jose, CA, USA). Each experiment was done in triplicate and repeated at least twice.

**Metabolite detection assay**

Cells were trypsinized and lysed in extraction buffer followed by centrifugation at 20 000 rpm for 10 min. The same amounts of lysates were taken for the detection of the listed metabolite below. AMP, ADP, ATP, GTP, GSH, GSSG, and NAD^+^ and NADH were detected using AMP assay kit (Solarbio® BC1024), ADP assay kit (Merck® MAK081), ATP content assay kit (Solarbio® BCBC0305), GTP assay (Shanghai Jianglai industrial® JL46555), GSH assay kit (Solarbio® BC1175), GSSG assay kit ((Solarbio® BC1180), NAD^+^ and NADH assay kit (Beyotime® S0175) according to instruction of the manufacturers, respectively. All experiments were performed at least three times and the data were normalized by the cell numbers or protein content.

**Quantitative real-time PCR**

TRIzol (Invitrogen, Carlsbad, CA, USA) was used using according to the instructions to extract total RNA from tissues or cells. Reverse transcription was conducted using total poly (A)-tailed RNA, reverse transcription primers and ImPro-II reverse transcriptase (Promega, Madison, WI, USA) according to manufacturer’s instructions. Fast Start Universal SYBR Green Master Mix (Rox) (Roche Diagnostics GmbH Mannheim, Germany) was used to conduct quantitative real-time PCR (qRT-PCR) as described by the manufacturer. KEGG term enrichment analysis of down or up regulated genes were performed.

**Western blot**

Cells or tissues were homogenized, then lysed with a RIPA lysate (Beyotime Biotechnolog, P0013B) containing protease inhibitor and 1mM PMSF. The bicinchoninic acid (BCA) method was used to determine the total protein concentration. An appropriate amount of proteins was mixed with loading buffer, and the mixture was boiled for 5 min to denature the protein. The proteins were sequentially electrophoresed, transferred and blocked. The corresponding primary antibodies were added and incubated overnight at 4 °C. After three washes with TBST (20 mM Tris-HCl, 150 mM NaCl, 0.1% Tween®20), the corresponding secondary antibodies were added and incubated for 1 hour at room temperature. After washing with TBST three times, the reaction bands were visualized using chemiluminescence agents and imaged in a BioImaging Systems instrument. The intensities of the bands were quantified by using ImageJ software (Maryland, USA, https://imagej.nih.gov/ij/). ImageJ was used to compare the density (aka intensity) of bands on western blot as follows: (1) Open the image file using File>Open in ImageJ; (2) The gel analysis routine requires the image to be a gray-scale image; (3) Choose the Rectangular Selections tool from the ImageJ toolbar, and draw a rectangle around all the gel bands; (4) Go to Analyze>Gels>Plot Lanes to draw a profile plot of each gel band; (5) The profile plot represents the relative density of the contents of the rectangle over each lane. The intensity should be subtracted by background signal.

**MTT assay**

The MTT assay was used according to the manufacturer’s instructions to evaluate the proliferation of target cells. Living cells can reduce exogenous MTT to formazan, which creates a purple sediment in dimethyl sulfoxide (DMSO). The OD of this sediment, which is proportional to the number of proliferating cells, was measured at 540 nm using a spectrophotometer.

**Plate colony forming assay**

The cells (density of 5 × 10^2^) were seeded in six-well plates and cultured for two weeks for colony forming assay. Cells were twice washed using PBS, fixed using acetic acid/methanol (1:3, v/v), and crystal violet stained (Sigma, China). A microscope (Olympus IX81, Japan) was used to count colonies.

**Cell Invasion and migration assay**

Transwell chambers (8 μm of pore size, BD Biosciences, USA) coated with matrigel were used to detect cell invasion and not coated with matrigel were used to detect cell migration. HCC cells were seeded on the top of matrix in the upper chamber, and the bottom chamber was filled with medium containing serum. After 24 hours, the cells that migrated into the coating layer of matrigel were fixated with paraformaldehyde, then stained with crystal violet. Cells were observed, counted and photographed under a microscope.

**Immunohisochemistry**

Hepatocellular carcinoma tissue and heterogeneous tumor tissue sections tissues were fixed with formalin and embedded in paraffin. Tissue sections were de-waxed in xylene and rehydrated using an ethanol graded series. Endogenous peroxidase activity was quenched by immersing sections for 30 minutes in 0.3% peroxidase-methanol solution. Antigen retrieval was carried out by pre-treating sections for 15 minutes at 100°C in citric acid buffer. Hybridization for four hours with primary antibody at 1:100 dilution was carried out, then sections were treated with the UltraVisionQuanto Detection System HRP DAB Kit (Thermo Scientific) according to manufactures instructions. Re-staining with hematoxylin was conducted and an Olympus BX51 microscope was used to take micrographs. Ten high power fields (×200) images were randomly selected and cancer cells were counted to investigate the number of ABHD2-positive cells.

**Spheroid formation assay**

A total of 1000 CC cells were plated in ultralow attachment plates. The cells were cultured for 10 days in DMEM/F12 medium (Invitrogen, Shanghai, China) supplemented with 4 mg/mL insulin (Sigma, Shanghai, China), B27 (1:50, GIBCO, Shanghai, China), 20 ng/mL EGF (Sigma, Shanghai, China) and 20 ng/mL basic FGF (Sigma, Shanghai, China). For the serial passaging of primary spheres, these were collected, dissociated with trypsin, resuspended in DMEM/F12 medium with the above supplements, and plated to generate secondary spheroids. The number of spheres was counted under the microscope, and the data are expressed as the mean ± SD of triplicate wells within the same experiment.

**RNA-Seq**

As described in the manufacturer's procedure, total RNA was isolated and purified by using TRIzol (Life, cat.265709, CA, USA). After the quality inspection of Agilent 2100 Bioanalyzer (Agilent, cat.G2939AA, CA, USA) and NanoPhotometer® (Implen, cat.N60, Munich, Germany), mRNA with poly(A) is purified from 1μg total RNA using VAHTS® mRNA Capture Beads with Oligo (dT) (Vazyme, cat.N401-01, Nanjing, China) through two rounds of purification. Subsequently, mRNA fragment was interrupted using VAHTS® Universal V6 RNA-seq Library Prep Kit (Vazyme, cat.NR604, Nanjing, China) under 94℃ for 8 min and reversed transcription into cDNA, which would be used to synthesize U-labeled second-stranded DNAs. An A-base was added to the blunt ends of each strand to ligase the indexed adapters that contains a T-base at the tail end. After UDG enzyme treatment of the U-labeled double-strand DNA, size selection was performed with VAHTS® DNA Clean Beads (Vazyme, cat.N411, Nanjing, China). Then, the ligated products were amplified with PCR as the following conditions: initial denaturation at 98℃ for 5 min; 12-17 cycles of denaturation at 98℃ for 10 sec, annealing at 60℃ for 30 sec, and extension at 72℃ for 30 sec; final extension at 72℃ for 5 min. The average insert size of cDNA library was 280±80 bp. After purification by VAHTS® DNA Clean Beads (Vazyme, cat.N411-02, Nanjing, China), quality control of concentration and fragment size is performed by Agilent 2100 Bioanalyzer (Agilent, cat.G2939AA, CA, USA) and Qubit assay tubes (Life, cat. 1604220, CA, USA). At last, we performed the 2×150bp paired-end sequencing (PE150) on an Illumina Novaseq™ 6000 system (Illumina Corporation, San Diego, USA) following the vendor's recommended protocol by Guangzhou Huayin Health Medical Group CO.,Ltd. (Guangzhou, China).

**Chromatin immunoprecipitation (ChIP)**

ChIP assays were performed by using the EZ-ChIP™ Chromatin Immunoprecipitation Kit (Millipore, Bedford, MA, USA) following the manufacturer's protocol. Briefly, protein and DNA were cross-linked using 1% formaldehyde for 10 min. Cell lysates were sonicated to obtain DNA fragments, which were subjected to IP with primary antibody or negative control IgG. Purified DNA was analyzed by qRT-PCR using SYBR Green Master Mix (Promega, Beijing, China). Relative enrichment values were calculated by normalizing the results to the input values and expressed relative to the values obtained with normal IgG.

**Bioinformatics Analysis**

Data quality control: Raw data was filtered using Cutadapt (https://cutadapt.readthedocs.io/en/stable/, v1.16) and a self-made program for removing adaptor contamination and low quality reads, respectively. rRNA contamination was filtered by Bowtie2 (http://bowtie-bio.sourceforge.net/ bowtie2/index. shtml, version: bowtie2, v2.3.3.1).

Genome alignment: Clean reads were mapped to the genome (for example: Homo sapiens) using TopHat (http://ccb.jhu.edu/software/tophat/index.shtml，v2.1.1).

Quantitative and differential analysis of gene expression: The expression level of mRNA were calculated using RSEM (RNA-Seq by Expectation Maximization) (v1.3.1) by normalized to FPKM (Fragments Per Kilobase Per Million reads). The differentially expressed mRNAs were screened using edge R package (https://bioconductor. org/packages/release/bioc/html/edgeR.html) in R. |log2Foldchange| ≥ 1and Pvalue < 0.05 was considered significant. GO and KEGG enrichment was completed using clusterprofiler package in R, subsequently. STRING[1] and Cytoscape 3.7.1[2] were used for network analysis and visualization of transcription factors.

Metabolic pathway analysis was carried out in the Pathway Analysis module of MetaboAnalyst 4.0[3]. All metabolites were normalized with total analyzed cell numbers for analysis. For metabolite clustering analysis, average linkage hierarchical clustering was determined in MetaboAnalyst 4.0 by using Euclidian distance as a similarity metric.

**Oligonucleotides for Qrt-PCR**

| **Name** | **Sequence** |
| --- | --- |
| β-actin-F | AACAGTCCGCCTAGAAGCAC |
| β-actin-R | CGTTGACATCCGTAAAGACC |
| Myc-F | CCCTATTTCATCTGCGACGAG |
| Myc-R | GAGAAGGACGTAGCGACCG |
| wnt9b-F | ACCTGAAGCAGTGTGACCTAC |
| wnt9b-R | GCTCCTGCCTGAACTGGAA |
| fzd9-F | TTGCTCTATTATTTCGGGATGGC |
| fzd9-R | CAGGACCACGATAGTTTTGAGTG |
| Prkcb-F | ATGAGTTCGTCACGTTCTCCT |
| Prkcb-R | CCATACAGCAGCGATCCACAG |
| ODC-F | TGTATCTGCTTGACATTGGTGGTG |
| ODC-R | CAGGAAGATACTATGTCGCATCAGC |
| HK2-F | TGCCACCAGACTAAACTAGACG |
| HK2-R | CCCGTGCCCACAATGAGAC |
| GLUT1-F | CTGCAACGGCTTAGACTTCGAC |
| GLUT1-R | TCTCTGGGTAACAGGGATCAAACA |
| TRIM32-F | CCGGGAAGTGCTAGAATGCC |
| TRIM32-R | CAGCGGACACCATTGATGCT |
| GAPDH-F | GGAGCGAGATCCCTCCAAAAT |
| GAPDH-R | GGCTGTTGTCATACTTCTCATGG |

**Reagents**

| **Reagents** | **Company** | **Identifier** | **Country** |
| --- | --- | --- | --- |
| SAM | Solarbio | IA0080 | China |
| Methionine | Solarbio | IM0550 | China |
| Serine | Solarbio | IS0640 | China |
| Folate | Sigma | F8758 | USA |
| DMSO | Solarbio | D8371 | China |
| Sorafenib | Solarbio | S5080 | China |
| Sirtinol | Solarbio | IS1890 | China |
| SAH | Sigma | A9384 | USA |
| NAM | aladdin | [A117203](https://www.aladdin-e.com/zh_cn/a117203.html) | China |
| FIDAS-5 | MCE | HY-136144 | USA |
| CHX | MCE | HY-12320 | USA |
| MG132 | MCE | [HY-13259](https://www.medchemexpress.cn/MG-132.html) | USA |

**References**

1. Szklarczyk D, Gable AL, Nastou KC, Lyon D, Kirsch R, Pyysalo S *et al.* The STRING database in 2021: customizable protein-protein networks, and functional characterization of user-uploaded gene/measurement sets. Nucleic Acids Res. 2021; 49: D605-D12.

2. Otasek D, Morris JH, Boucas J, Pico ARDemchak B. Cytoscape Automation: empowering workflow-based network analysis. Genome Biol. 2019; 20: 185.

3. Chong J, Wishart DSXia J. Using MetaboAnalyst 4.0 for Comprehensive and Integrative Metabolomics Data Analysis. Curr Protoc Bioinformatics. 2019; 68: e86.

**
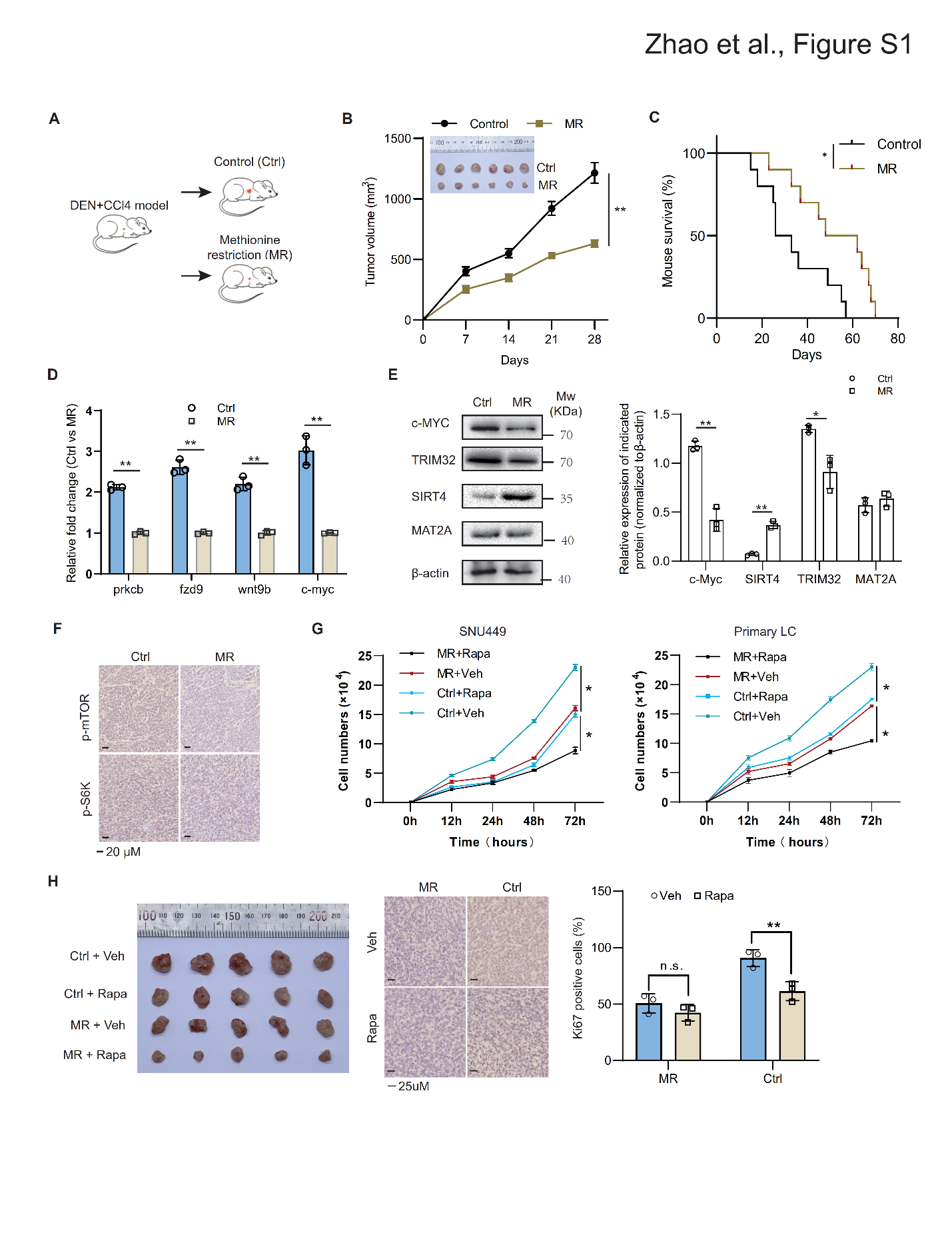
Additional Figures**

**Fig.S1.** **mTOR senses the availability of methionine.** (**A)** Schematic design of HCC DEN+CCl_4_ model (n = 6 mice per group). (**B)** Growth curves and images of tumours. (**C)** Survival analysis of mice under control or methionine restriction conditions. (**D)** Relative amounts of mRNA from MR or Ctrl liver samples analyzed by RT-qPCR. **(E)** Western blot analysis of selected proteins compared Ctrl to MR liver samples. Quantification was displayed in the right panel. (**F)** IHC showing activation of mTOR (phosphorylated mTOR: p-mTOR; phosphorylated p70 S6 kinase on serine 389: p-S6K) in liver tissues from Ctrl and MR mice. (**G)** Growth curves of SNU449 (left panel) and primary liver cells (right panel) treated with vehicle (Veh) or rapamycin (Rapa) under control or methionine restriction conditions. (**H)** Representative images, IHC analysis of Ki67, and quantification of Ki67-positive cells of mouse allograft tumours shown in **Fig. 1H**. Data are means ± SEM. Group differences were analyzed by two-tailed Student’s t test (B, D, E), two-way ANOVA followed by Tukey’s multiple comparison test (G, H) or log-rank test (C)(*p<0.05, **p<0.01). Abbreviations: n.s., not significant. Ctrl, Control and MR, Methionine restriction.


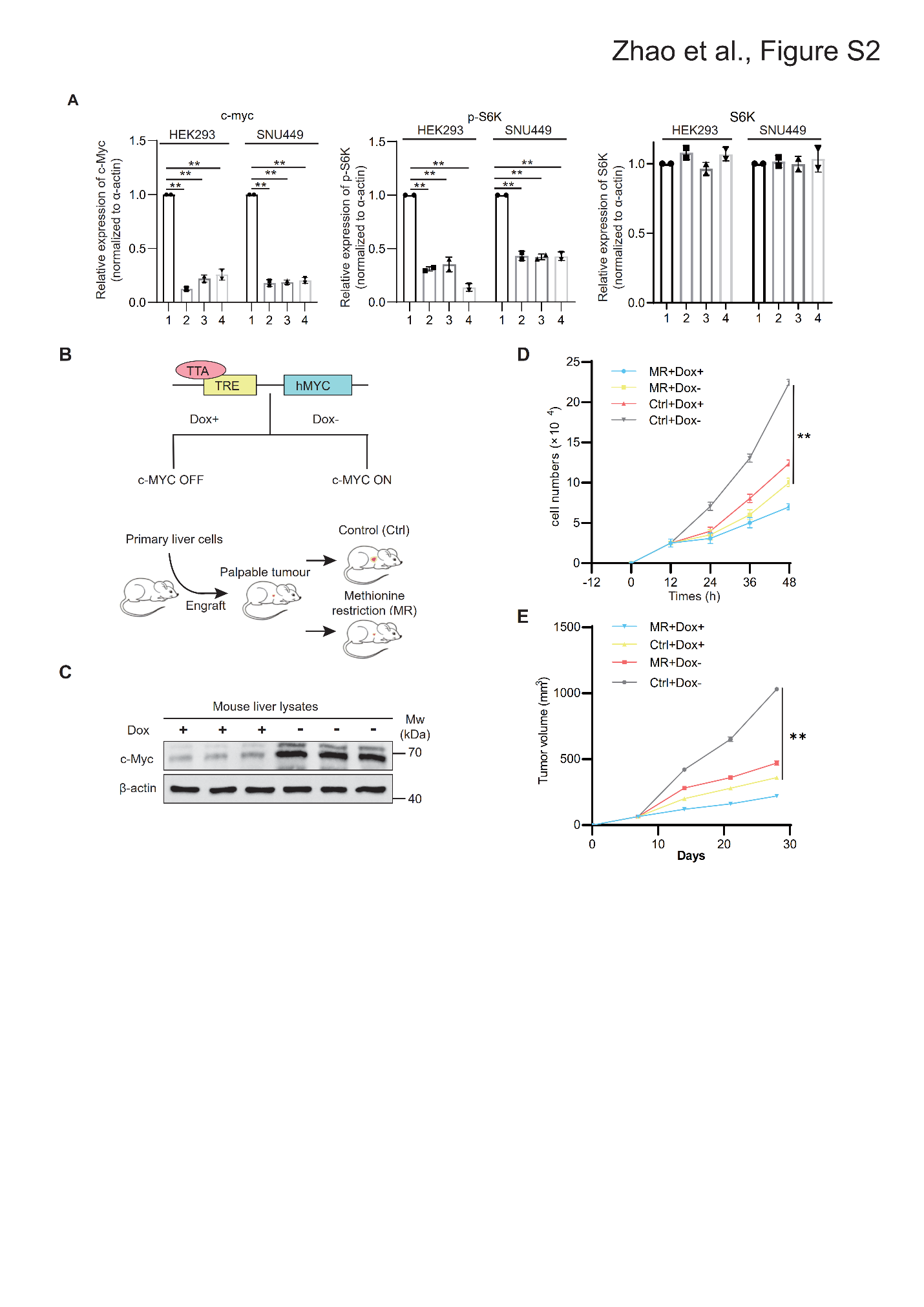
**Fig. S2.** **mTORC1 signaling pathway mediated Myc expression to promote HCC tumorigenesis.** (**A)** Western blot analysis of indicated proteins in the presence or absence of rapamycin (20 nM) when HEK293 or SNU449 cells were grown in Medium with or without methionine. Bar graphs showed the image quantifications of indicated proteins. (**B)** The cells were exposed to vehicle or 500 ng/mL doxycycline (DOX) under control or methionine restriction conditions for 96 h prior to analysis. Primary liver cells were injected subcutaneously into livers of nude mice. When tumour size reached ~65mm^3^, mice were fed with control or MR diets with or without DOX, and tumour growth was followed until tumour size reached 1 cm^3^. At the end of experiment, tumour lysates were subjected to immunoblotting with anti-Myc. (**C)** Immunoblot showing Myc protein level in primary liver cells expressing an inducible control or Myc shRNA. (**D)** Cell proliferation rate of primary liver cells described in (**B)**. (**E)** The tumour growth rate of mice described in (**B).** n = 5 mice per group. Data are means ± SEM. Group differences were analyzed by two-tailed Student’s t test (A) or two-way ANOVA followed by Tukey’s multiple comparison test (D, E) (**p<0.01). Abbreviations: n.s., not significant.


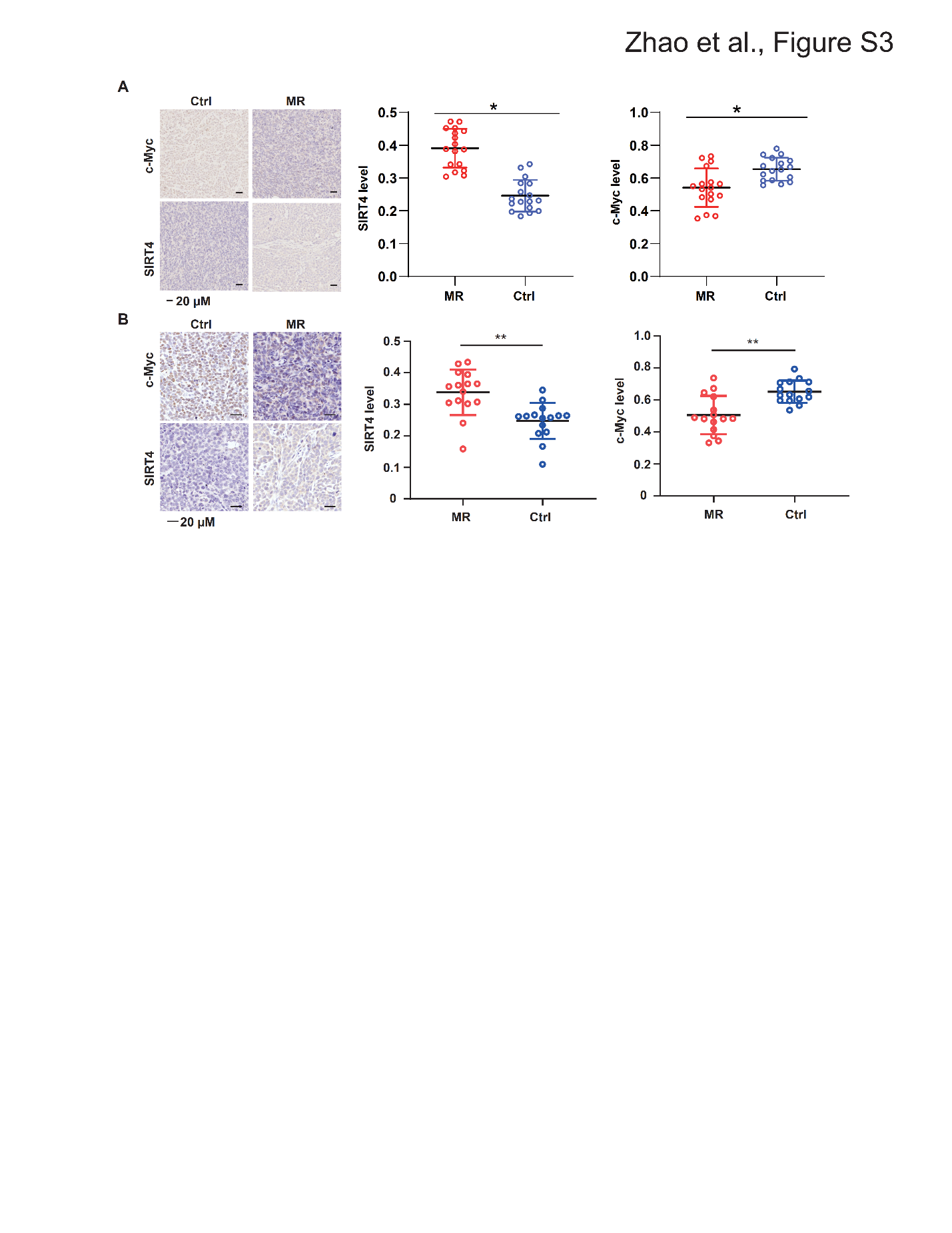


**Fig. S3.** **Negative correlation of MYC and SIRT4 expression.** Representative images of IHC staining for MYC and SIRT4 from Ctrl or MR treated mice liver tissue in the PDX (**A**) or DEN model (**B**). The intensities of Myc and SIRT4 shown in (**A-B**) were quantified using HistoQuest software. Data are means ± SEM. Group differences were analyzed by two-tailed Student’s t test (*p<0.05, **p<0.01).


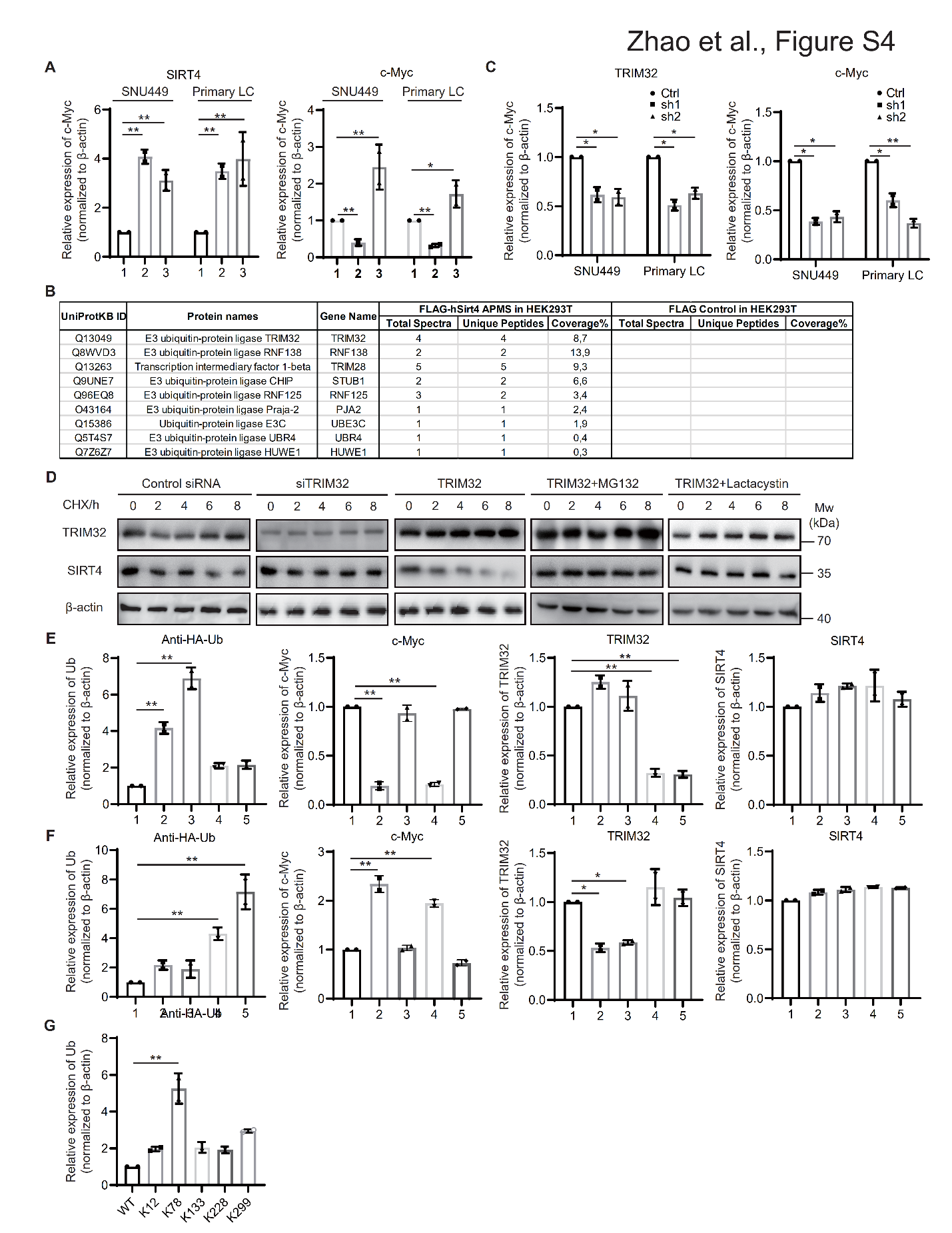


**Fig. S4. Myc regulates SIRT4 stability through proteasome degradation.** (**A)** Bar graphs showed the image quantifications of indicated proteins in **Fig. 2A**. (**B)** List of SIRT4-binding E3 ligases identified by IP and MS analysis in HEK293 cells (Anderson, et al. 2017). (**C)** Bar graphs displayed the image quantifications of indicated proteins in **Fig. 2C**. (**D)** Western blot analysis of indicated proteins under various conditions. TRIM32 was knockdown with shRNA or forced overexpressed in SNU449 cells with or without treatment with proteasome inhibitor MG132 or lactacystin. After being treated with protein synthesis inhibitor cycloheximide (CHX; 50 μg/ml), the above cells were collected at the indicated hours. (**E)** Bar graphs displayed the image quantifications of indicated proteins in left panel of **Fig. 2E**. (**F)** Bar graphs displayed the image quantifications of indicated proteins in right panel of **Fig. 2E**. (**G)** Bar graphs displayed the image quantifications of indicated proteins in **Fig. 2G**. Data are means ± SEM. Group differences were analyzed by two-tailed Student’s t test (*p<0.05, **p<0.01).

**
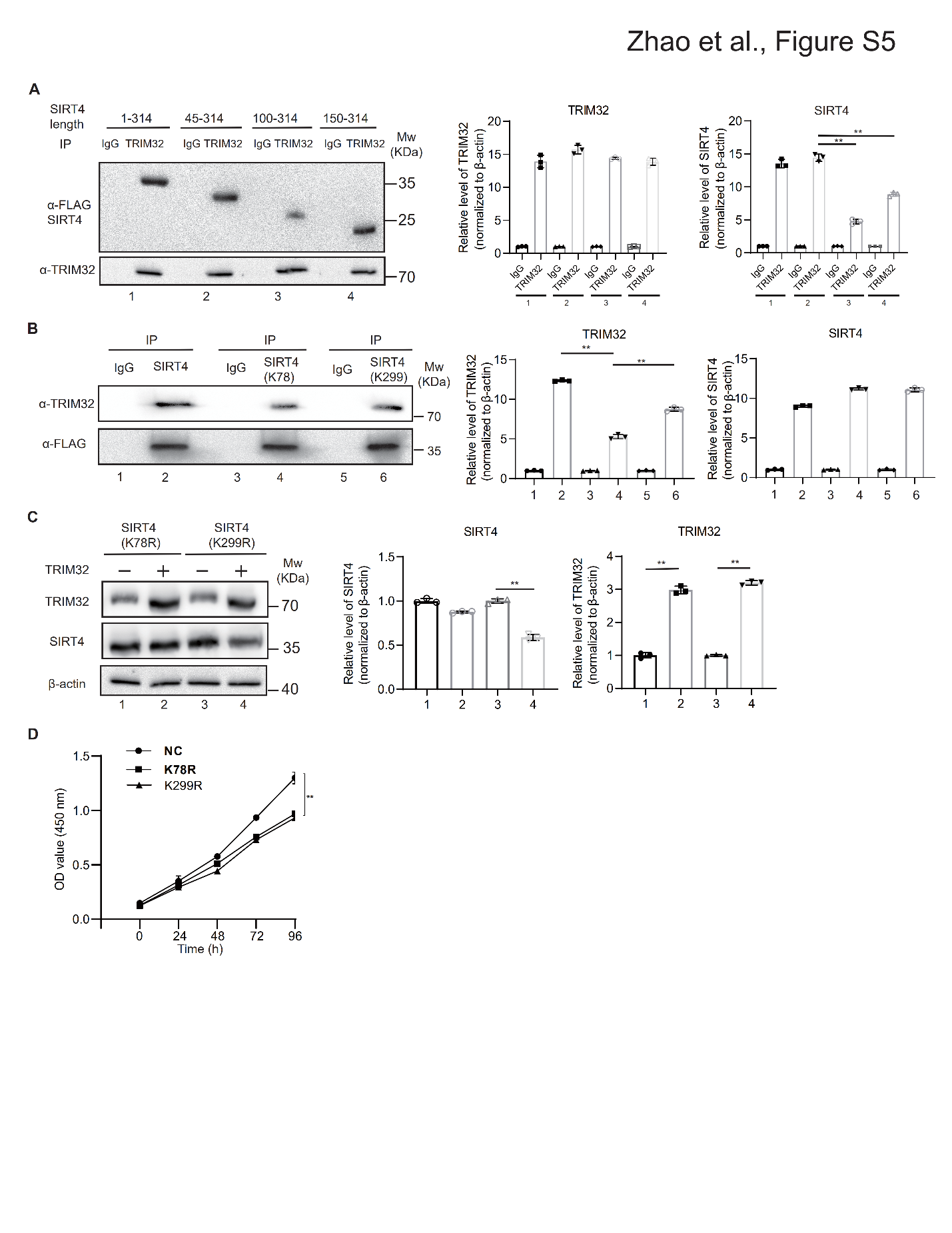
**

**Fig. S5. Characterization of the interaction between SIRT4 and TRIM32. (A)** TRIM32 interacts with WT or truncated SIRT4. FLAG-SIRT4 or truncated FLAG-SIRT4 with different length was expressed in HEK293 cells. HEK293 cell lysates were immunoprecipitated (IP) with control IgG, or anti-TRIM32 antibodies, and then the precipitated proteins were detected by anti-TRIM32 or anti-FLAG antibodies, respectively. Quantification was shown in the right panel. **(B)** FLAG-SIRT4 or FLAG-SIRT4 mutant (K78R or K299R) was expressed in HEK293 cells. HEK293 cell lysates were immunoprecipitated (IP) with control IgG, or anti-FLAG antibodies, and then the precipitated proteins were detected by anti-TRIM32 or anti-FLAG antibodies, respectively. Quantification was shown in the right panel. **(C)** FLAG-SIRT4 mutant (K78R or K299R) was expressed in HEK293 cells with or without overexpressing TRIM32. The cell lysates were taken for western blotting analysis of TRIM32 or FLAG-SIRT4 mutant expression. Quantification was shown in the right panel. **(D)** Cell proliferation rate of HEK293 cells expressing FLAG-SIRT4 mutant (K78R or K299R). The HEK293 cells was taken as the control. Data are means ± SEM. Group differences were analyzed by one-way ANOVA followed by Tukey’s test (**A**, **B, D**), or two-tailed Student’s t test (**C**) (**p<0.01).


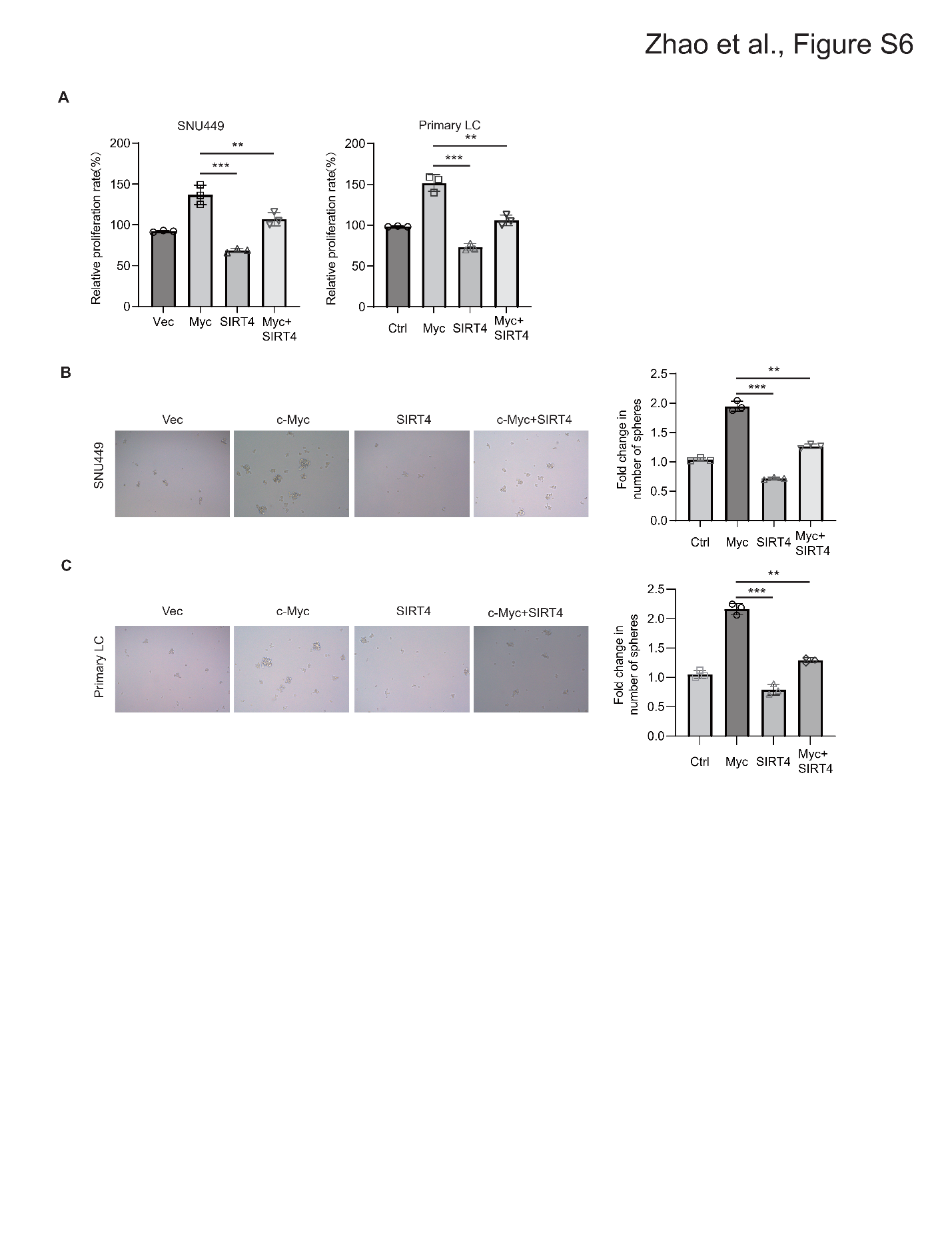
**Fig. S6. SIRT4 suppresses Myc promoted HCC tumorigenesis.** (**A)** Relative proliferation rates of SNU449 or primary liver cells expressing Myc, SIRT4 alone or together. (**B)** The effects of Myc and SIRT4 on cell stemness in SNU449 cells. (**C)** The effects of Myc and SIRT4 on cell stemness in primary liver cells. All experiments were repeated at least three times, and representative data are shown. Group differences were analyzed by two-tailed Student’s t test (**p<0.01, ***p<0.001).


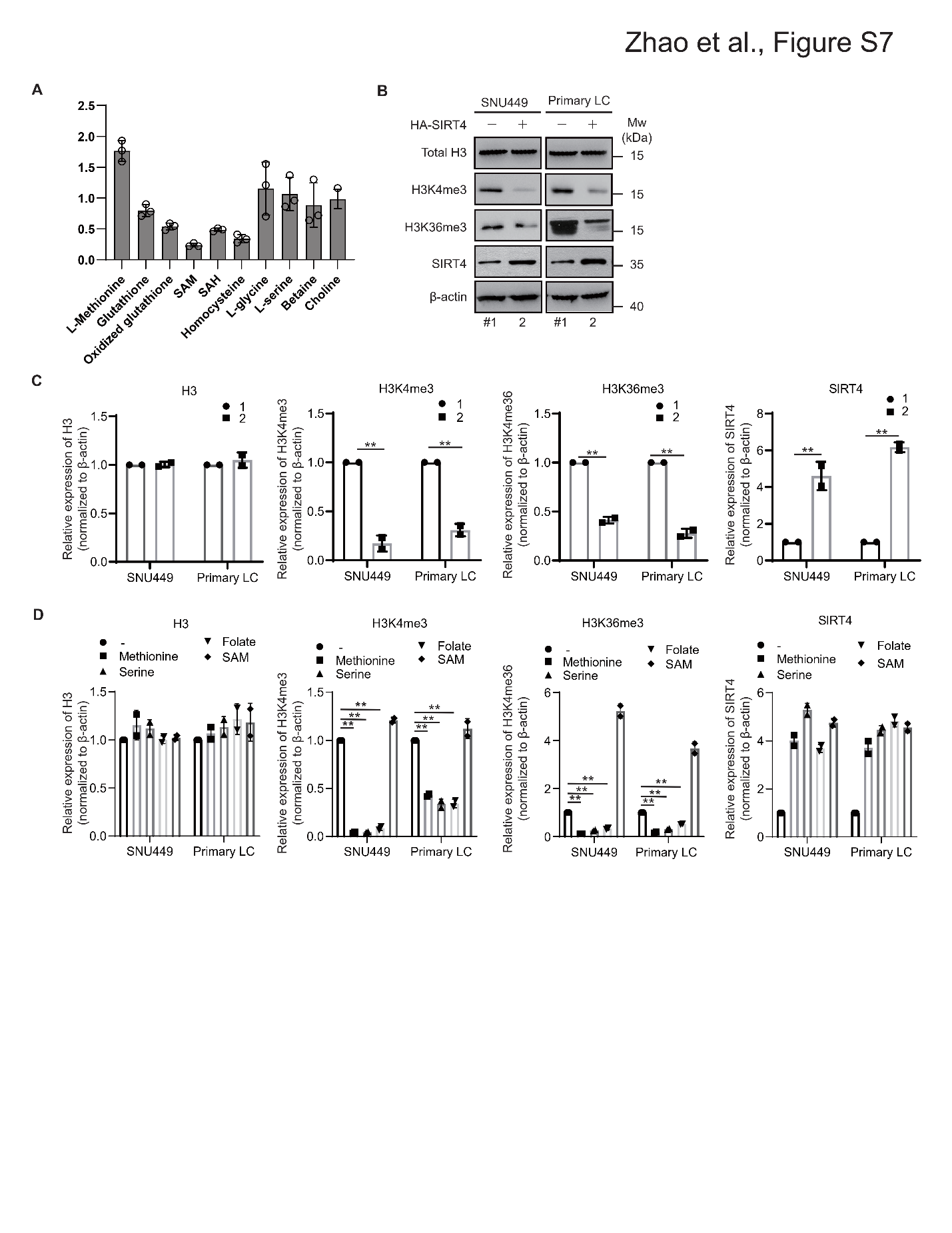
**Fig. S7. The effect of SIRT4 on histone methylation through regulating methionine metabolism.** (A**)** Metabolites related with methionine cycle were compared between control (Ctrl) and SIRT4 overexpressed cells. (**B)** Levels of modified histones in the control cells or SIRT4 overexpression cells. Histone H3 was used as a loading control. (**C)** Bar graphs displayed the image quantifications of indicated proteins shown in **(B)**. (**D)** Bar graphs displayed the image quantifications of indicated proteins in right panel of **Fig. 4E**. Group differences were analyzed by two-tailed Student’s t test (**p<0.01, ***p<0.001).


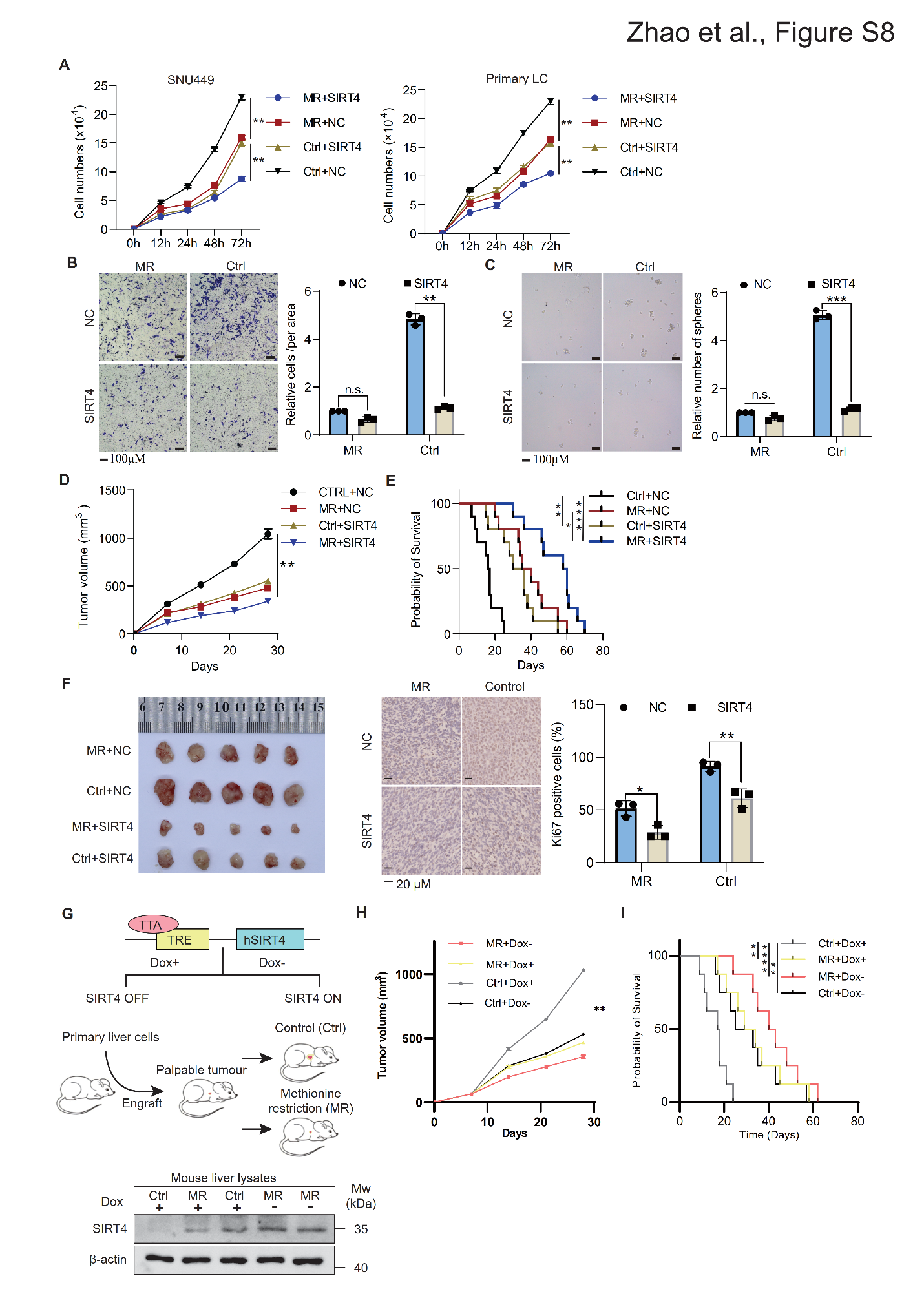


**Fig. S8. The effect of SIRT4 retards cancer progression through regulating methionine metabolism.** (**A)** Cell growth rates of SNU449 or primary liver cells with SIRT4 overexpression or not (NC) under control or methionine restriction conditions. (**B-C)** Migration and sphere formation efficiency of SNU449 with SIRT4 overexpression or not (NC) under control or methionine restriction conditions. Quantification were shown in the right panel. (**D-E)** Tumour volumes and survival analysis of mouse allografts implanted with SNU449 cells described in (**A)**. n = 5 mice per group. (**F)** Representative images, IHC analysis of Ki67, and quantification of Ki67-positive cells of mouse allograft tumours using SNU449 cells described in (**A)**. (**G)** Immunoblot showing SIRT4 protein level in primary liver cells expressing an inducible control or SIRT4 shRNA. The cells were exposed to vehicle or 500 ng/mL doxycycline (DOX) under control or methionine restriction conditions for 96 h prior to analysis. Primary liver cells were injected subcutaneously into livers of nude mice. When tumour size reached ~65 mm^3^, mice were fed with control or MR diets with or without DOX, and tumour growth was measured until tumour size reached 1 cm^3^. At the end of experiment, tumour lysates were subjected to immunoblotting with anti-SIRT4. n = 5 mice per group. (**H)** Cell proliferation rate of primary liver cells described in (**G)**. (**I)** The tumour growth rate of mice described in (**G).** Data are means ± SEM. Group differences were analyzed by two-way ANOVA followed by Tukey’s multiple comparison test (A,B,C,D,F,H) or log-rank test (E, I)(*p<0.05, **p<0.01). Abbreviations: n.s., not significant. Ctrl, Control and MR, Methionine restriction.


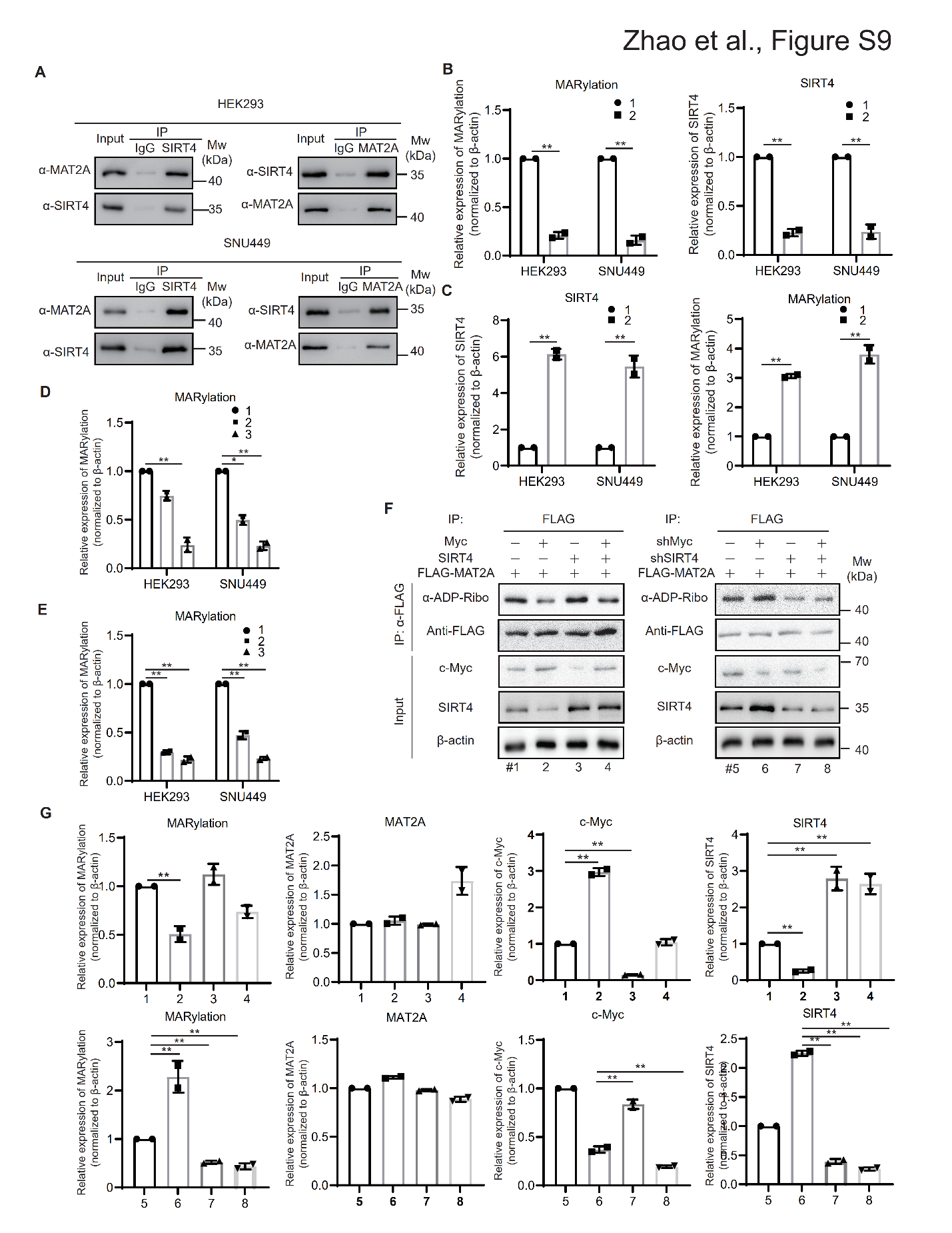


**Fig. S9. SIRT4 regulates MARylation of MAT2A.** (**A)** SIRT4 interacts with MAT2A in vivo. Total SNU449/HEK293 cell lysates were immunoprecipitated (IP) with control IgG, anti-SIRT4 or anti-MAT2A antibodies, and then precipitated proteins were detected by anti-MAT2A or anti-SIRT4 antibodies, respectively. (**B)** Bar graphs displayed the image quantifications of indicated proteins in right panel of **Fig. 5B**. (**C)** Bar graphs displayed the image quantifications of indicated proteins in right panel of **Fig. 5C**. (**D)** Bar graphs displayed the image quantifications of indicated proteins in right panel of **Fig. 5D**. (**E)** Bar graphs displayed the image quantifications of indicated proteins in right panel of **Fig. 5E**. (**F)** Western blot analysis of MARylation level of MAT2A, Myc and SIRT4 expression. (**G)** Bar graphs displayed the image quantifications of indicated proteins shown in (**F)**. Data are means ± SEM. Group differences were analyzed by two-tailed Student’s t test (**p<0.01).


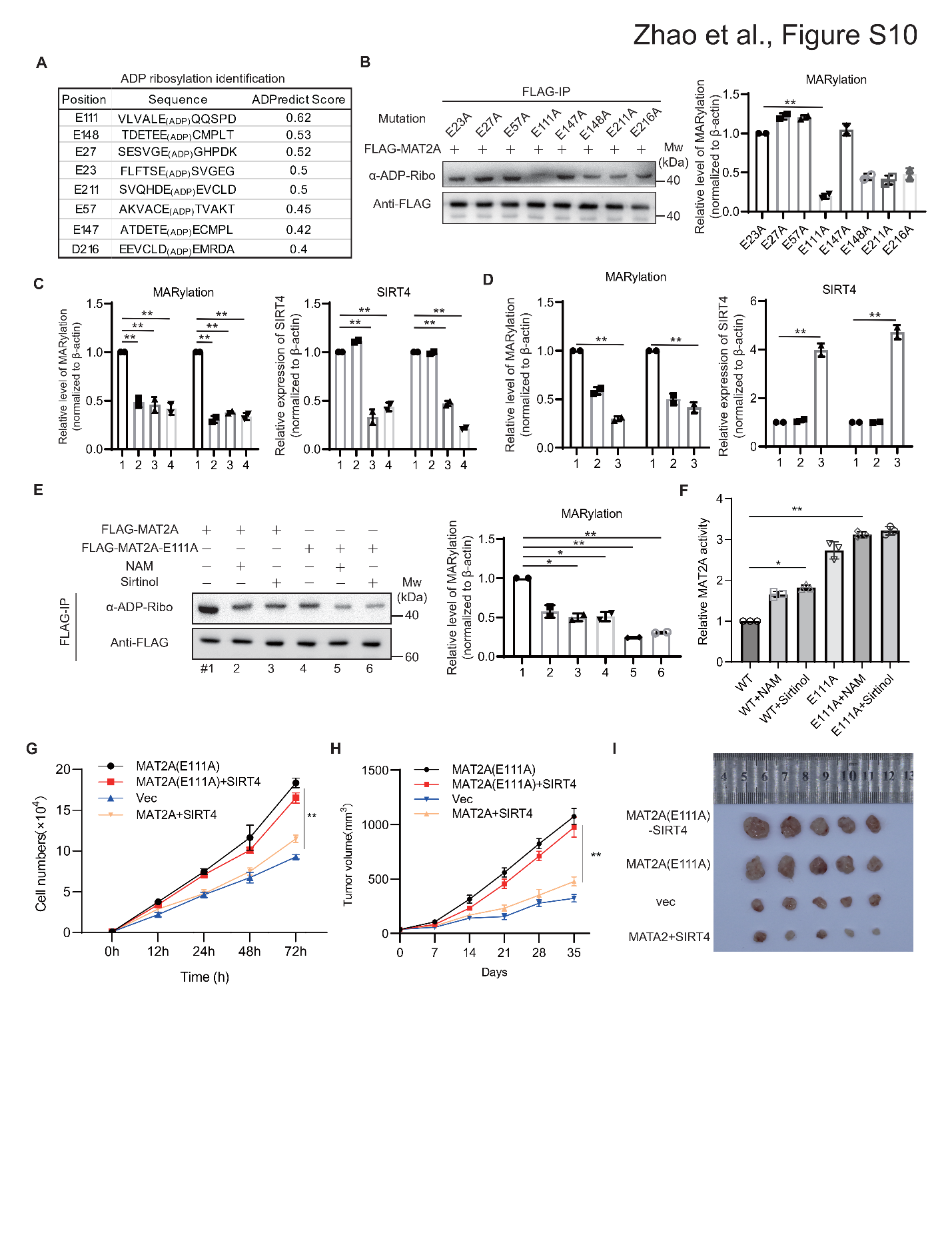


**Fig. S10. SIRT4 suppresses MAT2A promoted tumorigenesis.** (**A)** Possible ADP ribosylation sites of MAT2A was analyzed by ADPredict (<https://www.adpredict.net/index.php>). (**B)** Analysis of MARylation of individual MAT2A mutants. Bar graphs displayed the image quantifications of indicated proteins. (**C)** Bar graphs displayed the image quantifications of indicated proteins in right panel of **Fig. 5H**. (**D)** Bar graphs displayed the image quantifications of indicated proteins in right panel of **Fig. 5I**. (**E)** SNU449 cells transfected with indicated plasmids were treated with or without nicotinamide (NAM) or Sirtinol. Proteins were immunoprecipitated for MARylation analysis. Bar graphs displayed the image quantifications of relative MARylation level. (**F)** Activity of WT or mutant MAT2A upon NAM or Sirtinol treatment. (**G)** MAT2A knockout SNU449 cells were infected with viruses expressing MAT2A/SIRT4, mutant MAT2A(E111A) or MAT2A(E111A)/SIRT4. Cell growth curves were measured by trypan blue counting. (**H)** Effect of SIRT4 on the MAT2A mediated tumour-forming abilities. SNU449 cells used in (**G)** were injected subcutaneously into nude mice. (**I)** Tumours were extracted and compared at the end of the experiments described in (**H)**. n = 5 mice per group. Data are means ± SEM. Group differences were analyzed by two-tailed Student’s t test (B,C,D,E,F) or two-way ANOVA followed by Tukey’s multiple comparison test (G,H) (*p<0.05, **p<0.01).


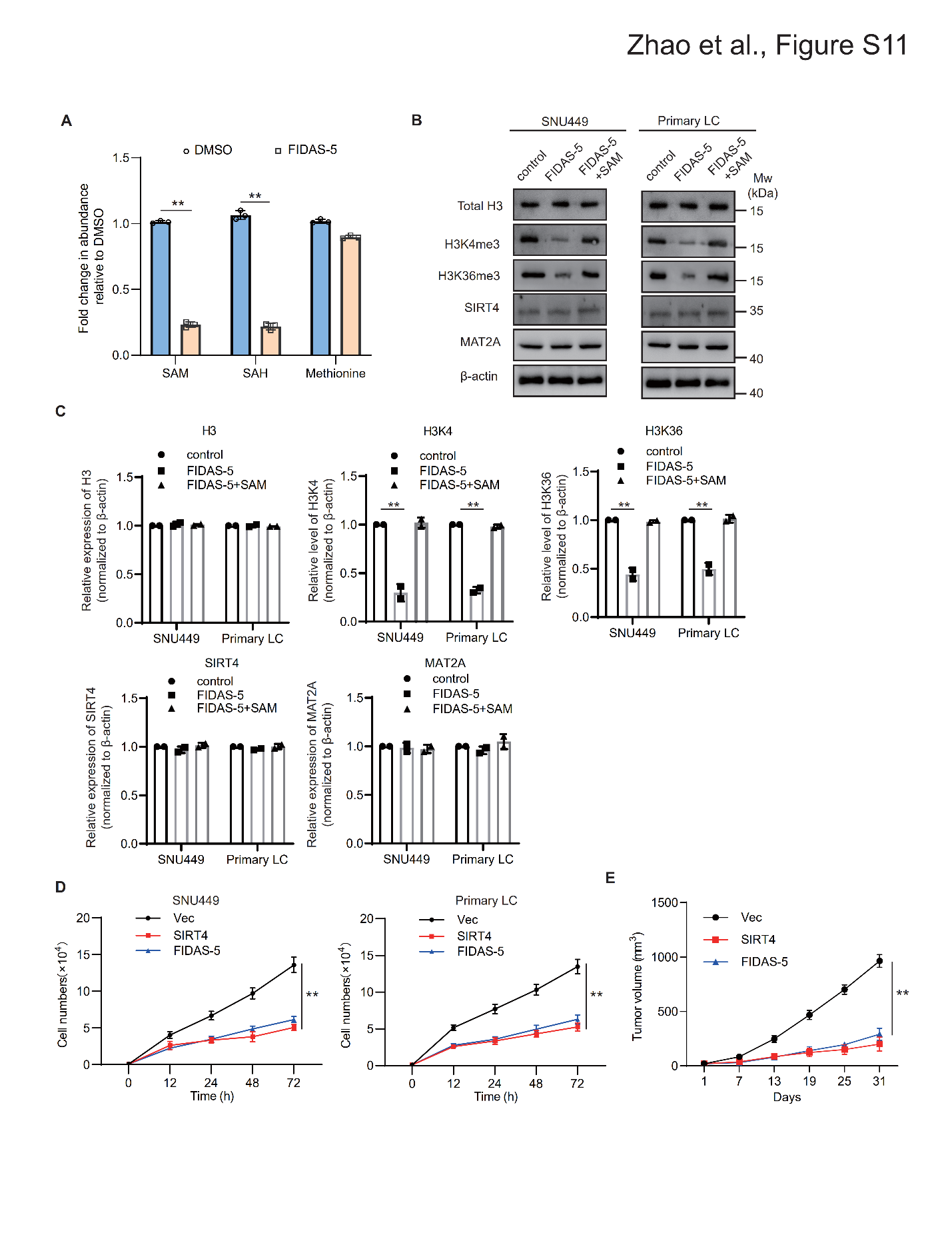
**Fig. S11. The MAT2A inhibitor FIDAS-5 abrogates HCC cell stemness.** (**A)** Abundances of intracellular primary methionine cycle metabolites were compared in the SNU449 cells with or without treatment FIDAS-5. (**B)** The effect of MAT2A inhibitor FIDAS-5 and SAM on the methylation level of histones in SNU449 and primary liver cancer (LC) cells. Histone H3 used as a loading control. (**C)** Bar graphs displayed the image quantifications of indicated proteins in (**B)**. (**D)** Cell growth curves of SNU449 or primary LC cells grown in the presence or absence of FIDAS-5 were measured by trypan blue counting. (**E)** SNU449 cells used in (**C)** were injected subcutaneously into nude mice. Effect of MAT2A inhibitor FIDAS-5 on the tumour-forming abilities was shown. n = 5 mice per group. Data are means ± SEM. Group differences were analyzed by one-way ANOVA followed by Tukey’s test (G,H) (**p<0.01).


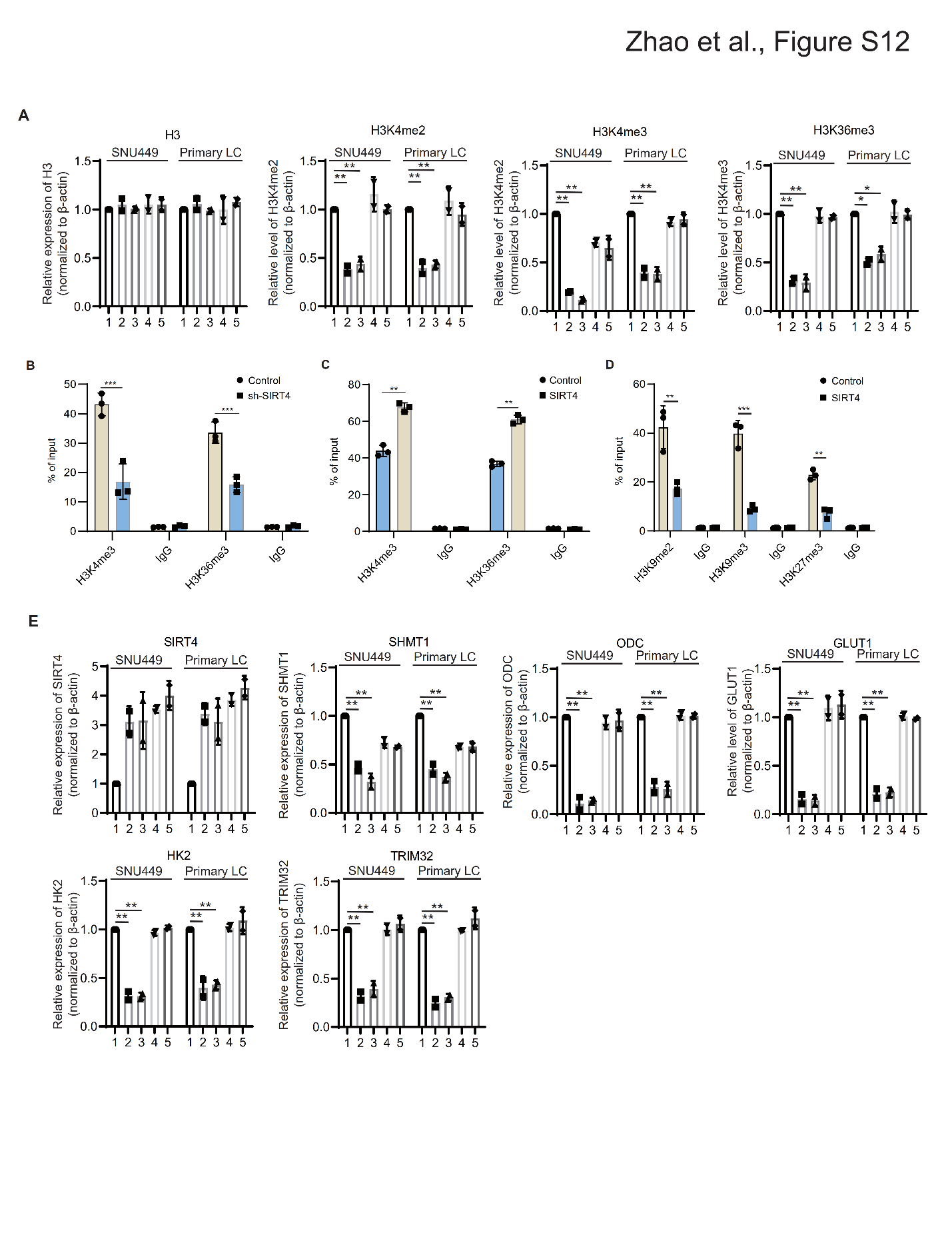
**Fig. S12. SIRT4 affects histone methylation and Myc mediated transcription.** (**A)** Bar graphs displayed the image quantifications of indicated proteins in **Fig.6A**. ChIP-qRT-PCR analysis determined the enrichment of H3K4me3 and H3K36me3 in the MYC promoter region following SIRT4 knockdown (**B**) or overexpression (**C**) in SUN499 cells. ChIP-qRT-PCR analysis determined the enrichment of H3K9me2, H3K9me3 and H3K36me3 in the MYC promoter region upon SIRT4 overexpression (**D**) in SUN499 cells. ChIP-qPCR was performed using IgG as a control. ChIP-qPCR was performed using IgG as a control. (**E)** Bar graphs displayed the image quantifications of indicated proteins in **Fig.6C**. Data are means ± SEM. Group differences were analyzed by two-tailed Student’s t test (*p<0.01, **p<0.01).


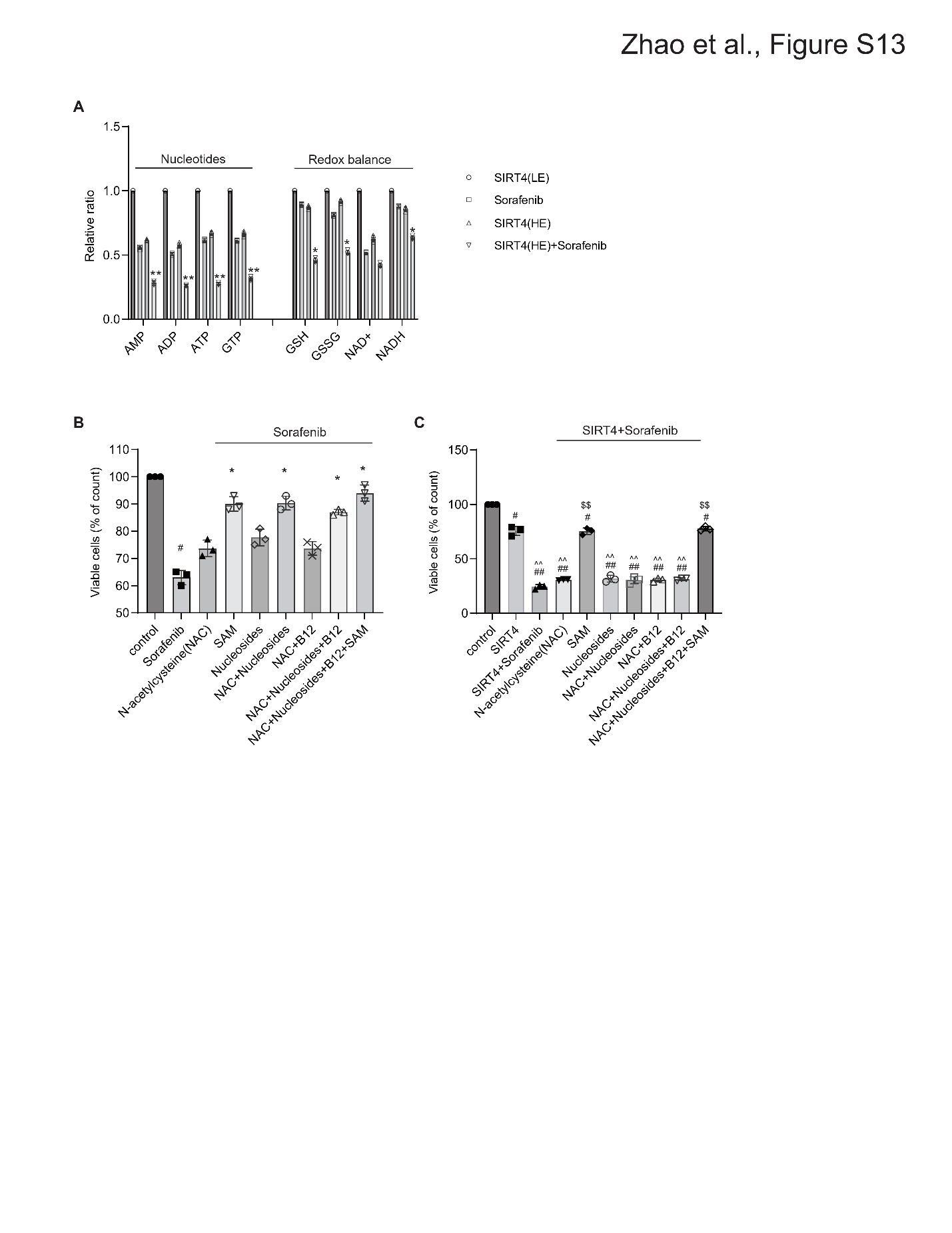


**Fig. S13. SIRT4 increases the sensitivity of HCC cells to chemotherapy.** (**a)** Relative intensities of metabolites related to nucleotide metabolism and redox balance in tumours. P values versus control was calculated by the two tailed Student’s t-test. Data is shown as Mean ± SEM. *P<0.05 versus Sorafenib treatment. (**b-c)** The rescue effect of NAC, SAM, on the inhibition of SNU449 cell proliferation mediated by Sorafenib (B) or SIRT4 and Sorafenib treatment (C). Data is shown as Mean ± SEM. P values versus control was calculated by the two tailed Student’s t-test. #P<0.05 versus control, ##P<0.01 versus control, and *P<0.05 versus Sorafenib treatment, ^^P<0.01 versus SIRT4 expression, $$ P<0.01 versus SIRT4+ Sorafenib treatment. Data are means ± SEM.


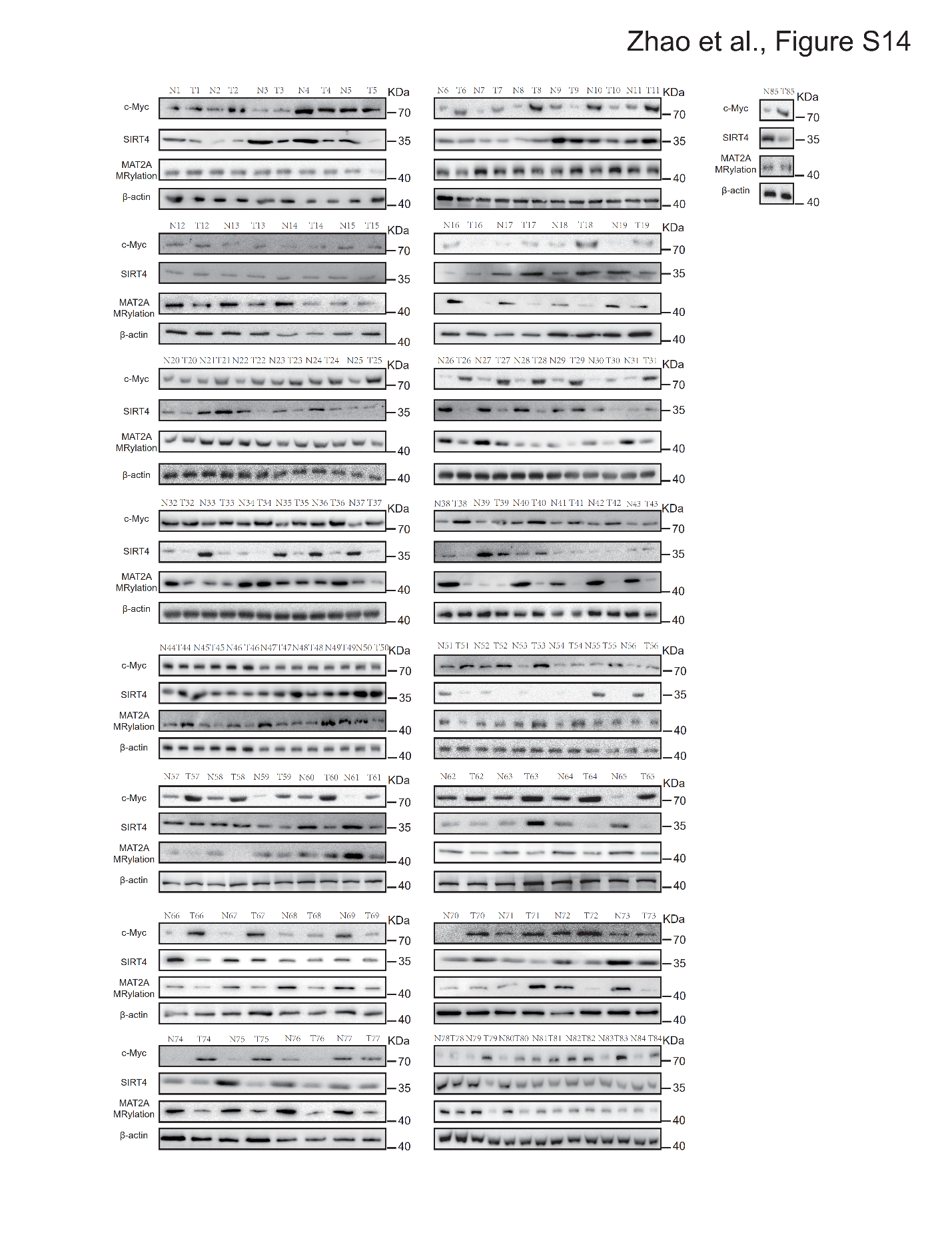


**Fig. S14. Comparison of c-Myc, MAT2A and MARylation level of MAT2A from** **clinical liver samples.** Protein levels of c-Myc, MAT2A and MARylation level of MAT2A were determined by western blot from 85 pairs of tumour tissues (N: Normal liver tissue; T: Tumour liver tissue). To measure the MARylation of MAT2A, we did immunoprecipitation of MAT2A from clinical liver samples first and then determined the MARylation level of MAT2A by WB using anti-pan-ADP-ribose binding reagent.
